# K-MARVEL: K-Mer-based antimicrobial resistance virtual exploration lab

**DOI:** 10.1038/s41467-026-77438-8

**Published:** 2026-09-03

**Authors:** Nirmal Singh Mahar, Sverre Branders, Manfred G. Grabherr, Ishaan Gupta, Rafi Ahmad

**Affiliations:** 1 Department of Biochemical Engineering and Biotechnology, Indian Institute of Technology Delhi, New Delhi, India; 2 Department of Biotechnology, University of Inland Norway, Hamar, Norway

**Keywords:** Software, Bacteria, Antimicrobial resistance

## Abstract

The rapid spread of antimicrobial resistance (AMR) necessitates new computational surveillance tools. Current methods for analysis of next-generation sequencing data have trade-offs: assembly-based approaches are computationally intensive, and direct long-read mapping is hampered by high error rates that obscure resistance-conferring mutations. Here, we present K-MARVEL, an open-source method that captures antimicrobial resistance genes (ARGs) and resistance-conferring mutations from both short- and long-read datasets. Operating in protein k-mer space, K-MARVEL tolerates nucleotide-level sequencing errors. We benchmarked K-MARVEL on 209 long-read and 205 short-read datasets across 22 bacterial species and achieved F1-scores of 0.976 (short-read) and 0.958 (long-read), outperforming assembly-based methods in speed and memory usage. K-MARVEL had higher F1-scores than seven widely used short-read-based ARG classifiers (0.979) and two widely used long-read-based classifiers (0.961) for homology-model-based ARGs. K-MARVEL can accurately identify both homologous ARGs and structural genes containing resistance-conferring mutations, including multiple variants, directly from raw sequencing data.

## Abstract (web summary)

Ahmad *et al* present K MARVEL, a scalable, flexible, memory-efficient solution for detection of antimicrobial resistance genes. K-MARVEL can handle data from both long- and short-read sequencing technologies, and resolve complex genomic scenarios, making it a potentially important tool for AMR surveillance.

## Introduction

Antimicrobial resistance (AMR) represents a critical and escalating global health threat, diminishing the efficacy of conventional antibiotics and complicating the treatment of bacterial infections^1^. The proliferation of AMR is primarily driven by the misuse and overuse of antimicrobial agents, which select for and facilitate the dissemination of antibiotic resistance genes (ARGs) within microbial populations^2^. Consequently, the precise identification and characterization of ARGs are fundamental to elucidating resistance mechanisms, monitoring their dissemination, and informing the development of novel therapeutic strategies^3,4^.

The advent of high-throughput sequencing technologies has revolutionized AMR surveillance, enabling the comprehensive detection of ARGs directly from complex metagenomic and clinical samples, without the limitations of conventional targeted molecular assays^5,6^. Standard bioinformatic methods for identifying ARGs in sequencing data typically involve either direct read alignment to curated ARG databases or a more computationally intensive assembly-first approach, in which reads are assembled into contigs prior to database mapping^5^. While the latter method mitigates challenges associated with short read lengths, its significant computational burden and time requirements often hinder rapid analysis.

Third-generation sequencing technologies, such as Oxford Nanopore Technologies (ONT), address the read-length limitation by generating long reads that can span complete genes in real-time, thereby simplifying read-based mapping^7,8^. However, the high error rate inherent to these technologies often obscures detection of specific point mutations, which is particularly crucial for identifying point mutations responsible for resistance in chromosomal genes (e.g., *gyrA*, *parC*, *rpoB*)^9^. Hybrid error-correction strategies, which leverage accurate short reads to polish error-prone long reads or de novo assembly, can improve accuracy. Nevertheless, these solutions are suboptimal for real-time applications due to the associated costs (hybrid sequencing), high computational complexity, and the requirement for sufficient sequencing depth^10,11^. However, the new R10.4.1 chemistry enables the generation of high-quality long reads that approach short-read accuracy, potentially improving assembly accuracy, bypassing the need for hybrid sequencing, and enhancing the detection of resistance genes and resistance-conferring mutations^12,13^.

Furthermore, in culture-free metagenomic samples, assembly is complicated by host DNA contamination, heterogeneous species abundance, and diverse microbial backgrounds^14,15^. Also, one major challenge in AMR surveillance is that current computational methods cannot simultaneously detect acquired resistance genes and resistance-conferring mutations without significant limitations. Methods like Resistance Gene Identifier (RGI) depend on prior genome assembly^16^, while tools like PointFinder require the user to specify the bacterial strain in advance, hindering the analysis of complex or unknown samples^17^.

To overcome these limitations, we present a novel, alignment-free method for the direct detection of ARGs and point mutations from raw sequencing reads. We introduce K-MARVEL (K-Mer based Antimicrobial Resistance Virtual Exploration Lab), an open-source software designed for efficient and accurate ARG profiling from both short- and long-read whole genome or metagenome sequencing data. K-MARVEL employs a specialized k-mer matching and extension strategy in protein space, enabling it to bypass the need for prior assembly or taxonomic classification. This enables simultaneous detection of acquired ARGs and resistance-conferring mutations in chromosomal genes.

We demonstrated that K-MARVEL outperforms assembly-dependent methods in computational efficiency while providing highly accurate results across multiple publicly available datasets spanning both long- and short-read technologies at varying sequencing depths. Furthermore, K-MARVEL achieved the highest classification accuracy when benchmarked against widely used read-based ARG classification tools for both short-read and long-read datasets.

## Results

We developed a novel method, K-MARVEL, for AMR detection and profiling directly from raw sequencing data, including both short-read and long-read datasets. This method employs a k-mer match-and-extend strategy in protein space, enabling it to capture both homologous ARGs and genes carrying resistance-conferring mutations. K-MARVEL is organized into four modules. K-MARVEL was further evaluated against other widely used read-based classifiers that either rely on read alignment or nucleotide k-mer-based detection. K-MARVEL consistently outperformed all evaluated tools in classification accuracy for both short-read and long-read datasets, demonstrating that protein-level detection substantially outperforms nucleotide-level approaches.

### Optimization of parameters for different sequencing technologies

To determine the optimal k-mer and extension size for both sequencing technologies, we performed a systematic grid search across the k-mer size ranging from 6 to 10 and the extension size ranging from 6 to 12.

We evaluated the tool’s classification performance on a set of 12 samples for which both short-read and long-read data, along with the reference-level assembly, were available in the public domain. For each combination, we aggregated the classification metrics across 12 samples, and the F1-score (Eq. 4) was used as the primary metric to determine the optimal combination of k-mer and extension size. The classification metric for each combination, by sequencing technology, is visualized in Supplementary Fig. 1. Based on the grid search, we found that the combination of 7 and 12 yielded the best performance on the long-read sequencing dataset, and the combination of 6 and 9 on the short-read data. Details on the ground truth set, captured ARGs, and classification metrics per sample are provided in Supplementary Data 1.

### Evaluation of classification performance on empirical datasets

After the parameter optimization, we expanded the tool’s testing on other publicly available datasets. We tested K-MARVEL on 209 long-read and 205 short-read datasets distributed across 22 bacterial species, the majority of which are on the pathogen priority list released by the World Health Organization (WHO)^18^ (Supplementary Fig. 2). All the datasets had reference-level genome assembly available, which was used to call the ARGs that served as the ground truth for the comparison (Refer to the methods for detailed information about the ground truth selection). The classification metrics for the comparison are shown in Tables 1 and 2 for long-read and short-read datasets, respectively. K-MARVEL achieved F1-scores of 0.958 and 0.976 for the long-read and short-read sequencing datasets, respectively, demonstrating its universal applicability across both short- and long-read sequencing technologies. Detailed information on the datasets used, ground truth, and observed ARGs is available in Supplementary Data 2 and 3 for the short-read and long-read datasets, respectively.

**Table 1:** Global Classification performance metrics of K-MARVEL for Long-read sequencing datasets with optimized k-mer and extension size (7, 12)

Number of datasets = 209			
Class designation		Ground truth	
		Positive	Negative
Predicted Class	Positive	(5626, **472**)	(309, **20**)
	Negative	(184, **16**)	(0, **0**)
Classification Metrics	Precision = (0.948, **0.959**)	Recall = (0.968, **0.965**)	F1-Score = (0.958, **0.962**)

Numerical values highlighted in **bold** represent the classification performance of K-MARVEL for capturing ARGs categorized under variant and overexpression models.

**Table 2:** Global Classification performance metrics of K-MARVEL for Short-read sequencing datasets with optimized k-mer and extension size (6,9)

Number of datasets = 205			
Class designation		Ground truth	
		Positive	Negative
Predicted Class	Positive	(5680, **482**)	(178, **16**)
	Negative	(97, **2**)	(0, **0**)
Classification Metrics	Precision = (0.969, **0.968**)	Recall = (0.983, **0.996**)	F1-Score = (0.976, **0.982**)

Numerical values highlighted in **bold** represent the classification performance of K-MARVEL for capturing ARGs categorized under variant and overexpression models.

### Benchmarking of K-MARVEL’s classification performance against other widely used read-based ARG classification methods

Next, we compared our tool’s classification performance with other widely used methods. For short-read datasets, we compared K-MARVEL’s classification performance against ARGprofiler, DeepARG, KARGA, KmerResistance, RGI-BWT, ShortBRED, and SRST2; for long-read datasets, we compared its performance against ABRICATE and KARGA. Except for K-MARVEL, all other tested tools did not support capturing ARGs categorized under the variant and overexpression models of the CARD database; benchmarking was performed using homology-model-based ARGs. The protein variant model comprises housekeeping genes or antibiotic targets that confer resistance in the presence of specific point mutations. In contrast, the protein overexpression model encompasses regulatory proteins where specific mutations lead to increased copy number or upregulated expression, resulting in clinical resistance.

**Fig. 1:**
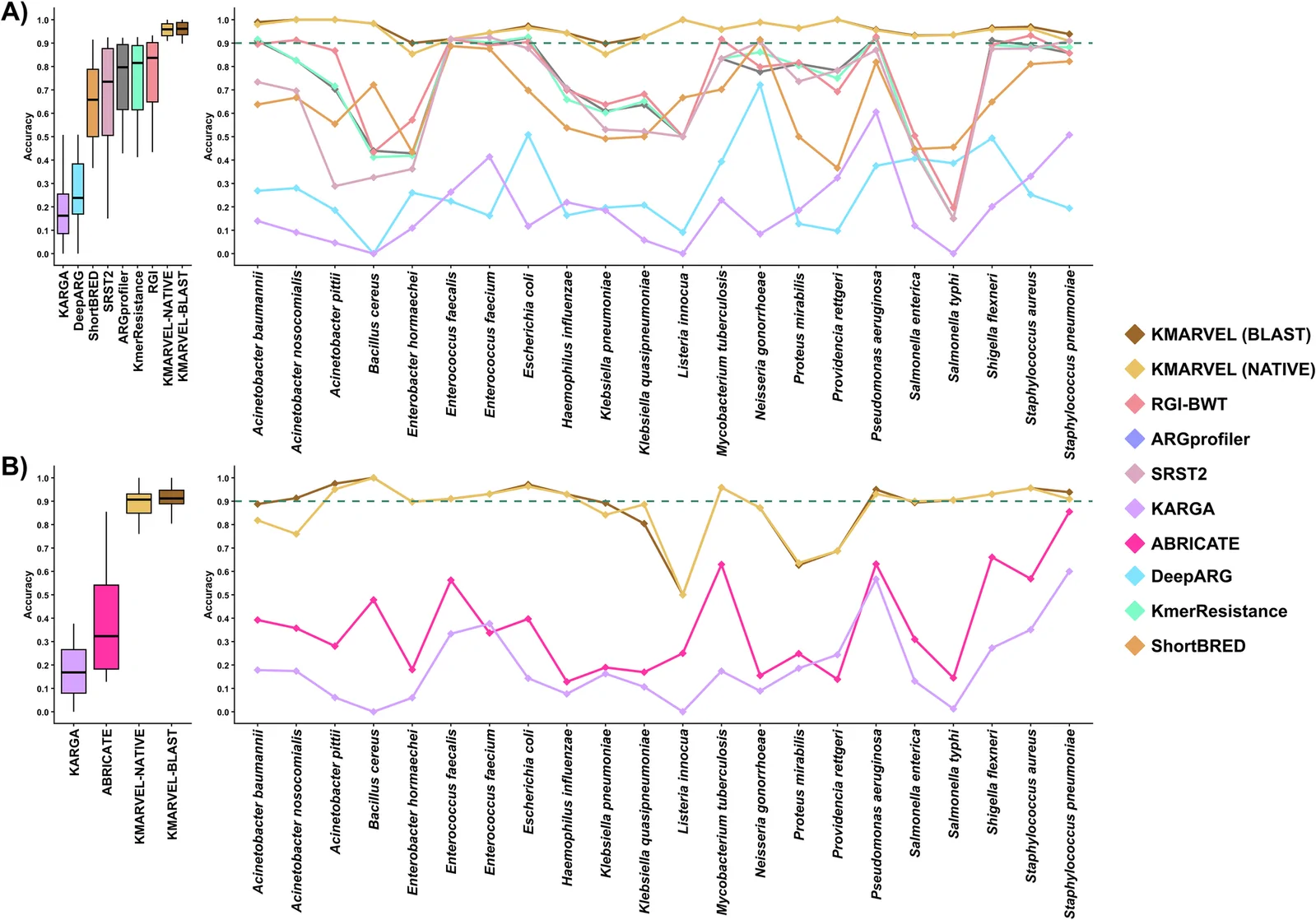
Comparison of the ARG classification performance between K-MARVEL and widely used read-based tools for both short-read and long-read datasets. **A** Box and line plot showing the accuracy of short-read ARG classification tools across different bacterial species datasets (*n* = 205). The x-axis represents classification tools in the box plot and bacterial species in the line plot; the y-axis represents classification accuracy in both plots. The horizontal dotted line in the line plot represents the accuracy of 0.9. The center line in the boxplot represents the median; the bounds of the box indicate the first and third quantiles (25 and 75 percentile); the whiskers extend to the most extreme data points within 1.5× interquantile range (IQR) from the box edges; points beyond the whiskers are plotted individually as outliers. The minima and maxima shown within the whisker range correspond to the lowest and highest non-outlier values, respectively. Overall, K-MARVEL consistently outperformed all other classification tools across all bacterial species. **B** Box and line plots showing the accuracy of long-read-based ARG classification tools across different bacterial species datasets (*n* = 209). The horizontal dotted line in the line plot represents the accuracy of 0.9. The center line in the boxplot represents the median; the bounds of the box indicate the first and third quantiles (25 and 75 percentile); the whiskers extend to the most extreme data points within 1.5× interquantile range (IQR) from the box edges; points beyond the whiskers are plotted individually as outliers. The minima and maxima shown within the whisker range correspond to the lowest and highest non-outlier values, respectively. As with short-read datasets, K-MARVEL demonstrated superior classification accuracy on long-read sequencing datasets.

Overall, K-MARVEL outperformed all other read-based classification tools for both short-read and long-read sequencing datasets. K-MARVEL (NATIVE) achieved a mean F1-score of 0.97 ± 0.02 (precision=0.967 ± 0.027; recall=0.982 ± 0.028), and K-MARVEL (BLAST) achieved a mean F1-score of 0.978 ± 0.017 (precision=0.971 ± 0.024; recall=0.986 ± 0.022) for short-read sequencing datasets. For long-read datasets, K-MARVEL (NATIVE) achieved a mean F1-score of 0.92 ± 0.08 (precision=0.897 ± 0.124; recall=0.965 ± 0.046) and K-MARVEL (BLAST) achieved a mean F1-score of 0.93 ± 0.08 (precision=0.905 ± 0.128; recall=0.97 ± 0.034). K-MARVEL also maintained classification accuracy (Eq. 5) ≥ 90% for 21 of 22 bacterial species with short-read sequencing data (Fig. 1A) and 13 of 22 with long-read sequencing data (Fig. 1B). Overall, K-MARVEL (BLAST) achieved an accuracy of 0.958 for short-read sequencing datasets and 0.925 for long-read sequencing datasets (Table 3). Detailed classification metrics for each tested tool, across each bacterial species and sequencing technology, are provided in Supplementary Data 4. Additionally, detailed information on the ground truth and ARGs captured by each tested tool is provided in Supplementary Data 5 and 6 for the short-read and long-read datasets, respectively.

**Table 3:** Global classification performance metrics of read-based ARG classification tools utilized in this study

Method	Sequencing Data	True Positive (TP)	False Positive (FP)	False Negative (FN)	Precision	Recall	F1-Score	Accuracy
SRST2	Short-Read	4455	698	839	0.864	0.842	0.853	0.744
ARGprofiler	Short-Read	4532	334	762	0.931	0.856	0.892	0.805
RGI-BWT	Short-Read	4641	434	653	0.914	0.876	0.895	0.810
KARGA	Short-Read	1438	2191	3856	0.396	0.272	0.322	0.192
**K-MARVEL** (**BLAST**)	**Short-Read**	**5208**	**140**	**86**	**0.974**	**0.984**	**0.979**	**0.958**
K-MARVEL (NATIVE)	Short-Read	5183	167	111	0.969	0.979	0.974	0.949
DeepARG	Short-Read	2925	3271	2369	0.472	0.552	0.510	0.341
ShortBRED	Short-Read	4282	1211	1012	0.779	0.810	0.794	0.658
KmerResistance	Short-Read	4529	331	765	0.932	0.855	0.892	0.805
ABRICATE	Long-Read	4524	7043	1272	0.391	0.781	0.521	0.352
KARGA	Long-Read	1652	2476	4144	0.400	0.285	0.333	0.200
**K-MARVEL** (**BLAST**)	**Long-Read**	**5624**	**284**	**172**	**0.952**	**0.970**	**0.961**	**0.925**
K-MARVEL (NATIVE)	Long-Read	5578	336	218	0.943	0.962	0.952	0.910

Best performing tool is highlighted in **bold**.

The method with the highest classification performance is highlighted in bold. K-MARVEL (NATIVE) represented the ARGs captured by K-MARVEL natively, whereas K-MARVEL (BLAST) represented the captured ARGs re-annotated using protein BLAST. Per species, classification metrics for each method are provided in Supplementary Data 4.

Since read-based classification methods were not designed to capture variant- and overexpression-model-based ARGs, we did not benchmark K-MARVEL’s classification performance for these ARGs against existing methods. However, we compared its performance against the ground truth obtained from the reference assemblies. Overall, K-MARVEL achieved an F1-score of 0.962 and 0.982 for long-read and short-read datasets, respectively (Tables 1 and 2).

We further investigated the false positives and negatives in the comparison of variant- and overexpression-model-based ARGs. The false negatives were either mis-assigned by K-MARVEL relative to the ground truth, or due to the sequencing data not sufficiently covering the target gene. False positives, however, were broadly classified into three categories. The first category contained the ARGs that confer resistance in the presence of multiple resistance variants. Since RGI does not support calling multiple resistance variants based on ARGs, these ARGs were not a part of the ground truth. The second category contained the ARGs with resistance-conferring mutations but with identity or coverage below the 90% cutoff. K-MARVEL, however, was able to reconstruct these genes, therefore keeping them in the final output file. The last category had the fewest false positives, emphasizing that these are rare events. Genes in this category were fragmented due to large insertions during assembly. Because of this, these genes were filtered out due to failing the coverage-based threshold (90%). However, K-MARVEL was able to reconstruct these fragmented genes because k-mers were available across the entire gene body.

Detailed information on false positives and negatives, categorized by sequencing technology, is available in Supplementary Data 7.

### Benchmarking of K-MARVEL’s computational performance against other widely used read-based ARG classification methods

**Fig. 2:**
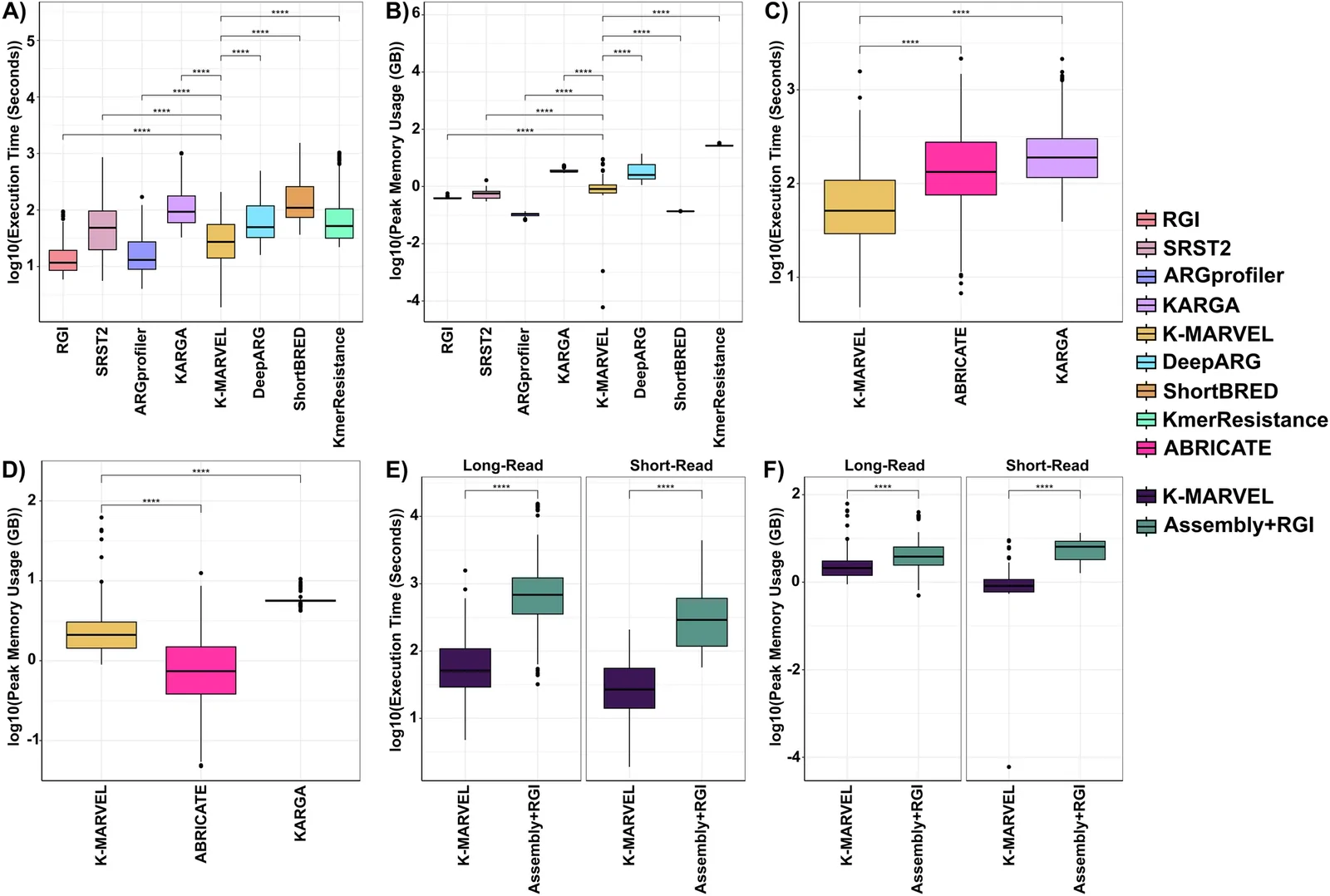
Comparison of the computational performance between K-MARVEL and read-based classification tools for both short and long-read datasets (*n* = 205, 209, respectively). All boxplots follow the same convention: the center line denotes the median; the box bounds represent the first and third quantiles (25 and 75 percentile); the whiskers extend to the minimum and maximum values within 1.5× interquantile range (IQR) from the box edges; points beyond the whiskers are plotted individually as outliers. All axes are log_10_ scaled and smaller values indicate better performance. Statistical significance (‘****‘, adjusted *p* value ≤ 0.0005) was assessed using a two-sided Wilcoxon rank-sum test with the Benjamini-Hochberg correction across all panels. **A** Boxplot showing the comparison of execution time (seconds) between K-MARVEL and other short-read-based classification tools. RGI + BWT was the fastest among all the compared tools, followed by ARGprofiler, then K-MARVEL. Adjusted *p* values for all pairwise comparisons versus K-MARVEL are as follows: RGI-BWT = 3.15 × 10^−17^, SRST2 = 6.40 × 10^−5^, ARGprofiler = 3.58 × 10^−13^, KARGA = 1.13 × 10^−35^, DeepARG = 3.36 × 10^−16^, ShortBRED = 2.46 ×  10^−44^, KmerResistance = 6.34 × 10^−15^. **B** Boxplot showing the comparison of peak memory usage (GB) between K-MARVEL and other short-read-based classification tools. Overall, ARGprofiler had the lowest memory footprint among all short-read-based classification tools. Adjusted *p* values for all pairwise comparisons versus K-MARVEL are as follows: RGI-BWT = 1.01 × 10^−66^, SRST2 = 9.82 × 10^−22^, ARGprofiler = 5.16 × 10^−67^, KARGA = 1.19 × 10^−61^, DeepARG = 7.99 × 10^−56^, ShortBRED = 3.40 × 10^−68^, KmerResistance = 3.92 × 10^−68^. **C** Boxplot showing the comparison of execution time (seconds) between K-MARVEL and other long-read-based classification tools. K-MARVEL was the fastest among the long-read-based classification tools compared. Adjusted *p* values for all pairwise comparisons versus K-MARVEL are as follows: ABRICATE = 1.31 × 10^−17^, KARGA = 2.04 × 10^−31^. **D** Boxplot showing the comparison of peak memory usage (GB) between K-MARVEL and other long-read-based classification tools. ABRicate showed the lowest memory footprint across all tested long-read classification tools. Adjusted *p* values for all pairwise comparisons versus K-MARVEL are as follows: ABRICATE = 1.48 × 10^−29^, KARGA = 6.80 × 10^−53^. **E** Boxplot showing the comparison of execution time (seconds) between the assembly-based approach and K-MARVEL, the adjusted *p*-value for this comparison is 9.38 × 10^−59^ and 1.95 × 10^−57^ for short-read and long-read data, respectively. **F** Boxplot showing the comparison of peak memory usage (GB) between the assembly-based approach and K-MARVEL, the adjusted *p*-value for this comparison is 4.58 × 10^−63^ and 4.72 × 10^−17^ for short-read and long-read data, respectively. K-MARVEL demonstrates significant reductions in execution time and memory consumption for both short-read and long-read datasets.

We further benchmarked K-MARVEL’s computational performance against other tested tools for both short-read and long-read datasets. All the tools were tested with 16 computation threads on a server equipped with AMD EPYC 7543 (32 cores) and 300 GB of RAM. For short-read sequencing datasets, RGI-BWT was the fastest among all tested tools with a median processing time of 11.68 seconds, closely followed by ARGprofiler with a median processing time of 13.1 seconds. Since both these tools used the KMA aligner^19^ for the read alignment step, they were expected to have a similar runtime profile. K-MARVEL followed RGI + BWT and ARGprofiler with a median processing time of 26.92 seconds (Fig. 2A). For long-read datasets, K-MARVEL required the least processing time, with a median execution time of 50.57 seconds (Fig. 2C). In terms of total memory footprint, ARGprofiler was the most memory-efficient short-read-based classification tool, with a median peak memory of 0.11 GB, followed by ShortBRED with a median peak memory of 0.13 GB. K-MARVEL exhibited the fourth-largest memory footprint of all short-read-based classification tools tested, with a median peak memory of 0.82 GB (Fig. 2B). For long-read datasets, ABRICATE had the smallest memory footprint, with a median peak memory of 0.74 GB, followed by K-MARVEL with a median peak memory of 2.07 GB (Fig. 2D). With respect to computational performance, K-MARVEL was not the most efficient among the evaluated read-based classification tools, which can be attributed to its k-mer match-and-extend strategy rather than read mapping. By contrast, K-MARVEL demonstrated the highest classification accuracy for both short-read and long-read datasets, surpassing all other tools.

We also compared the computational performance of K-MARVEL with the most widely used assembly-based approaches. We used Flye version 2.9.6^20^ to assemble the long reads, and SPAdes version 4.2.0^21^ with the ‘--isolate‘ parameter to assemble the short reads. RGI version 6.0.5 was then used to identify ARGs in the assembled contigs.

K-MARVEL performed significantly faster than the assembly-based approaches for both short- and long-read sequencing datasets (Fig. 2E), with a median execution time of 50.57 s for the long-read dataset, compared to 684.46 s for the assembly-based approach. For short-read datasets, K-MARVEL achieved a median execution time of 26.92 seconds, which was significantly lower than the assembly approach (290.44 s) (adjusted *p* value < 0.0005, Wilcoxon rank-sum test). The tool’s memory footprint was also significantly lower than that of the assembly approach for both short-read and long-read sequencing datasets (Fig. 2F). While processing short-read datasets, it maintained a median peak memory footprint of 0.82 GB, which was approximately 8 times lower than the memory footprint of the assembly-based approach (6.471 GB) (adjusted *p* value < 0.0005, Wilcoxon rank-sum test). In case of long-read datasets, K-MARVEL maintained a median peak memory footprint of 2.11GB, which was significantly lower than the assembly-based approach (3.84 GB) (adjusted *p* value < 0.0005, two-sided Wilcoxon rank-sum test with multiple testing correction via the Benjamini-Hochberg (BH) procedure).

Overall, in terms of execution time, K-MARVEL performed faster than the assembly-based approaches for both short and long-read datasets. The tool’s memory footprint was significantly lower than that of its counterpart for both short-read and long-read datasets. Based on these observations, it is safe to say that K-MARVEL is suitable for both long- and short-read datasets in terms of both classification accuracy and computational footprint, making it well-suited for processing large metagenomic datasets.

### Evaluation of K-MARVEL’s scalability for handling large-scale sequencing datasets

To evaluate the computational efficiency of K-MARVEL, we collected the runtime metrics of the tool across all tested empirical datasets with the optimized k-mer and extension size. The short-read datasets ranged from 0.79 million to 34.26 million reads, and the long-read datasets ranged from 4,174 to 2.61 million reads. Execution time was measured as the total wall-clock time (in seconds), and peak memory usage was recorded in GB. For short-read datasets, the execution time ranged from 6.7 to 208.49 s, while peak memory usage ranged from 0.54 to 9.1 GB. For long-read datasets, the execution time ranged from 4.78  to 1571.14 s, while peak memory usage ranged from 0.89 to 62.117 GB.

**Fig. 3:**
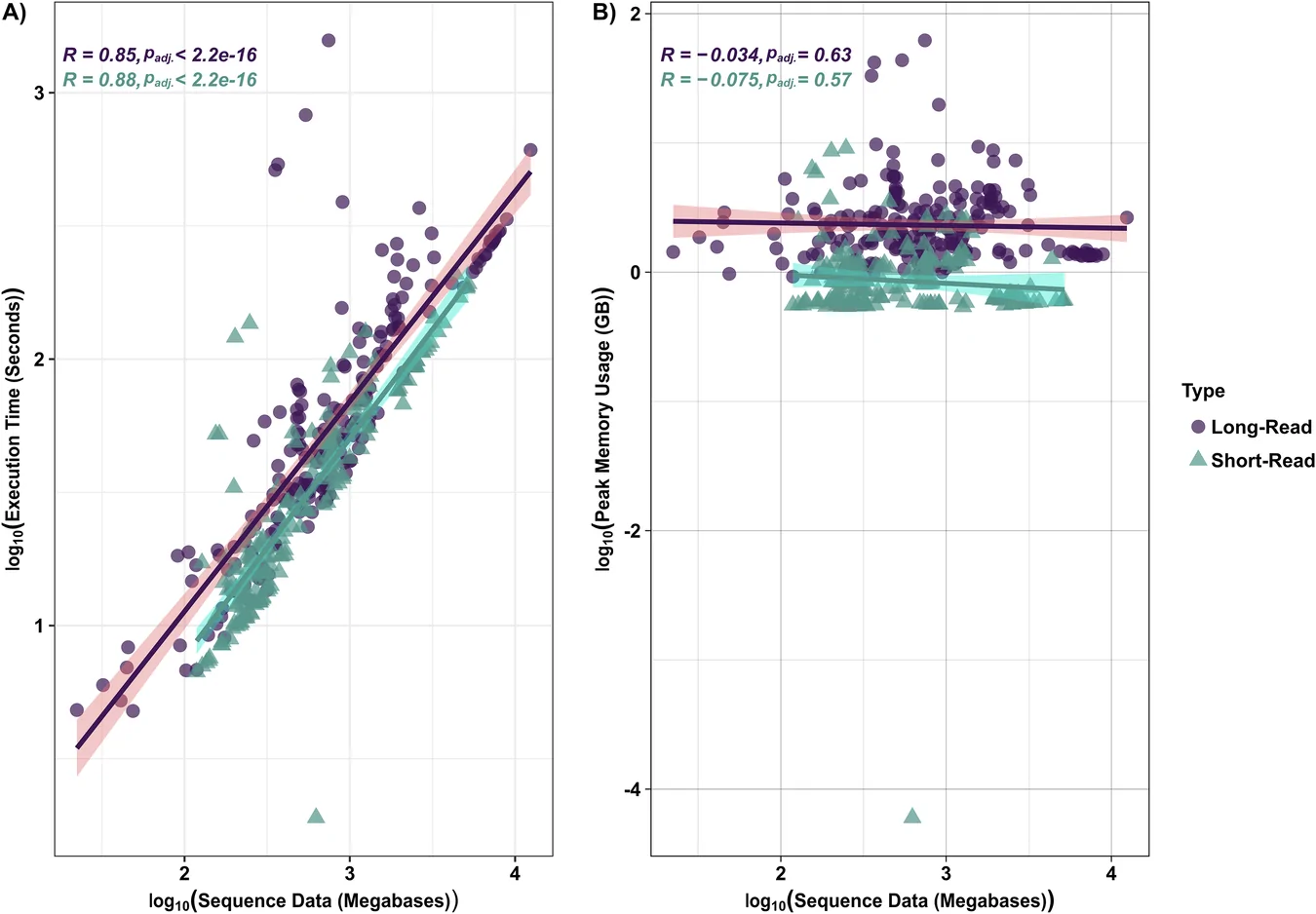
Computational performance and scalability of K-MARVEL. The run-time and peak memory usage of the tool were recorded across diverse sets of short-read (teal circles) (*n* = 205) and long-read (purple triangles) (*n* = 209) datasets. Both axes are scaled on a log10 scale to visualize performance across several orders of magnitude of sequence data. Pearson’s R (two-sided) was calculated to assess the relationship between the computational metric and the sequencing data, with multiple testing correction via the Benjamini-Hochberg (BH) procedure. **A** Execution time. For long-read datasets, the adjusted *p* value was 2.42 × 10^−60^ (95% CI: 0.809-0.884), and for short-read datasets, the adjusted *p*-value was 2.38 × 10^−66^ (95% CI: 0.839–0.904). **B** Peak memory usage. For long-read datasets, the adjusted *p* value was 0.625 (95% CI: −0.168 to 0.102), and for short-read datasets, the adjusted *p* value was 0.566 (95% CI: −0.209 to 0.062). The execution time scaled nearly linearly for both short- and long-read datasets, demonstrating the tool’s scalability. Memory consumption for both short-read and long-read datasets remains consistently low and stable regardless of the data size.

While comparing the execution time with respect to the dataset size (number of reads), we observed two distinct, linear performance profiles corresponding to the sequencing technology used (Supplementary Fig. 3). For short-read data, execution time showed a strong, significant correlation with the number of reads in the dataset (two-sided Pearson’s R = 0.87, *p*_*adj*._ < 0.001). This strong correlation suggests highly efficient, low-computational-cost processing of short-read sequencing data. In contrast, long-read sequencing datasets exhibited a relatively steeper linear relationship (two-sided Pearson’s R = 0.64, *p*_*adj*._ < 0.001). While the correlation was statistically significant, we observed greater variance in runtime for long-read datasets, likely due to the read-length-agnostic technology. We also assess the correlation between computational metrics and data size, measured by the total number of sequenced bases. The tool’s execution time scaled linearly with respect to the number of sequenced bases for both long-read (two-sided Pearson’s R = 0.85, *p*_*adj*._ < 0.001) and short-read (two-sided Pearson’s R = 0.88, *p*_*adj*._ < 0.001) datasets. This near-linear relationship indicates that the runtime scales (Fig. 3A). In terms of memory footprint, it showed no significant correlation in either the short-read (two-sided Pearson’s R = –0.075, *p*_*adj*._ = 0.28) or long-read datasets (two-sided Pearson’s R = –0.034, *p*_*adj*._ = *0.63*). The nearly flat regression lines and scattered data points suggest that memory consumption does not scale proportionally with dataset size and remains stable regardless of the dataset’s size for both short-read and long-read sequencing datasets (Fig. 3B).

Detailed information about the execution time, peak memory usage, and other computation metrics for each tested method is provided in the Supplementary Data 8.

## Discussion

In this study, we developed and validated K-MARVEL, a high-performance computational method for capturing the homologous resistant genes and structural genes with resistance-conferring mutations from both short-read and long-read sequencing data. A major challenge in long-read sequencing, particularly with Oxford Nanopore technology, is the prevalence of insertion–deletion (INDEL) errors arising from homopolymer regions and modified bases in neighboring k-mers^9,22^. These errors can lead to false detection of resistance-conferring variants, as it is difficult to distinguish between sequencing errors and true mutations. While short-read sequencing data is limited by read lengths ranging from 100 bp to 300 bp, leading to fragmented genome assemblies^23^. K-MARVEL addresses the challenges of both short- and long-read sequencing data by utilizing k-mers and performing all operations in protein space. This method avoids the assembly step, significantly reducing both execution time and memory usage. It is capable of detecting ARGs without relying on genome assembly, making it especially suitable for quick analysis in environments that use long-read sequencing, such as clinical metagenomics or real-time surveillance.

To assess K-MARVEL’s performance, we evaluated it on a large benchmarking dataset comprising 209 long-read and 205 short-read sequencing samples. These samples were distributed across 22 phylogenetically distant bacterial species, most of which are included on the WHO pathogen priority list. Furthermore, we ensured that most bacterial species had comparable numbers of long-read and short-read datasets to minimize discrepancies arising from differences in sequencing technology (Supplementary Fig. 4).

Across benchmarking datasets, K-MARVEL demonstrated superior computational efficiency compared with existing assembly-based methods for both short-read and long-read sequencing technologies. It achieved high F1-scores of 0.976 and 0.958 for short- and long-read sequencing, respectively, indicating a robust balance of precision and recall. A comparative evaluation against widely used read-based ARG detection tools further showed that K-MARVEL significantly outperformed existing methods in capturing homologous ARGs, achieving F1-scores of 0.979 (short-read) and 0.961 (long-read), compared with RGI-BWT (0.895, short-read) and ABRICATE (0.521, long-read). Furthermore, K-MARVEL maintained high performance in detecting variant- and overexpression-model-based ARGs, i.e., structural genes that confer resistance only in the presence of specific mutations. K-MARVEL achieved F1-scores of 0.982 for short-read datasets and 0.962 for long-read datasets, demonstrating its ability to identify both types of ARGs, homologous ARGs and structural genes with resistance-conferring mutations. Moreover, K-MARVEL can capture multiple resistance variants, a category that state-of-the-art assembly-based methods like RGI do not support.

In terms of computational performance, both ARGprofiler and RGI-BWT were the most resource-efficient methods for short-read sequencing data. K-MARVEL showed superior runtime performance on long-read sequencing datasets, though its memory footprint was relatively higher than that of most tested tools. Nevertheless, its computational footprint remained manageable on portable laptops. This is particularly relevant given the recent advancement of miniature, portable, point-of-care sequencing devices, which create a need for efficient, lightweight analysis tools. Given K-MARVEL’s computational performance on both short-read and long-read datasets, the tool can perform efficiently on modern portable laptop computers, making it well-suited for portable real-time surveillance and point-of-care clinical metagenomics.

K-MARVEL can also potentially reconstruct full-length resistance genes even when the genome is fragmented by using k-mers that cover the entire gene. This makes it possible to recover genes in regions that are hard to assemble, such as repetitive sequences. For example, in the *Shigella flexneri* AUSMDU00009397 genome, the *sul2* gene appears in two separate fragments (73.06% and 26.94%). These fragments were not recovered by assembly-based methods due to low coverage, but K-MARVEL was able to reconstruct the full gene. K-MARVEL may reconstruct genes that are actually disrupted in the genome, for example, by large structural changes within the gene. In these cases, the reconstructed sequence may not represent a functional or expressed gene. Overall, while K-MARVEL is useful for detecting ARGs in regions that are difficult to assemble, its results should be interpreted carefully and, where possible, supported by experimental data.

A recent innovation in nanopore sequencing is duplex basecalling, which leverages both the template and complementary strands to generate a consensus sequence, thereby significantly reducing the error rate^24,25^. However, duplex reads typically constitute only a small fraction of the total sequencing output compared to simplex reads^26^. The long-read datasets analyzed in this study comprised eight single-species duplex basecalled samples, with read counts ranging from 4,174 (~6x sequencing depth) to 13,090 ( ~ 29.6x sequencing depth). Despite this low read depth, K-MARVEL consistently maintained an F1-score > 0.91 across all duplex datasets. This observation is especially relevant to complex clinical samples, where high levels of host DNA contamination often yield very few microbial reads. In a clinical setting, reliably capturing the resistome at low coverage remains a major challenge^27,28^. However, our results demonstrate that K-MARVEL can recover ARGs even from a limited number of high-accuracy duplex reads. These findings suggest that continued improvements in duplex read yield, when combined with K-MARVEL, could substantially enhance resistome profiling in complex clinical samples with high host-genome contamination. Detailed information regarding the classification performance of K-MARVEL on duplex datasets is available in Supplementary Data 9.

Although K-MARVEL offers several benefits, it has certain limitations. Its fundamental approach of reference k-mer match-and-extend makes it heavily dependent on the reference gene database, which means it is not ideal for identifying completely new resistance determinants. Although several well-maintained ARG databases are available, such as MEGARes^29^, the national database of antibiotic-resistant organisms (NDARO)^30^, and ResFinder^31^, K-MARVEL’s default database is the Comprehensive Antibiotic Resistance Database (CARD)^32^. However, users can use any database as long as it follows a similar format to CARD. In the future, we plan to develop solutions to enable seamless integration of additional well-maintained databases, minimizing user intervention. K-Marvel is an assembly-free approach that is currently not supported for capturing the ARG’s pathogen of origin. Identifying the pathogen of origin will require separate genomic methods. Future work will focus on testing with complex clinical metagenomic samples and developing modules to identify the pathogen of origin linked to the AMR.

In conclusion, K-MARVEL represents a significant advance in reference-based ARG detection from metagenomic data. By operating in protein space and bypassing assembly, it uniquely combines high accuracy with low computational cost across both short- and long-read technologies. Its ability to capture multiple resistance variants, a feature not supported by current methods like RGI, highlights its potential to reveal previously overlooked resistance mechanisms. K-MARVEL offers a fast, flexible, and scalable solution for detecting both homologous ARGs and structural genes with resistance-conferring mutations directly from raw sequencing data. Its compatibility with both long- and short-read sequencing technologies, as well as AMR monitoring and detection.

## Methods

**Fig. 4:**
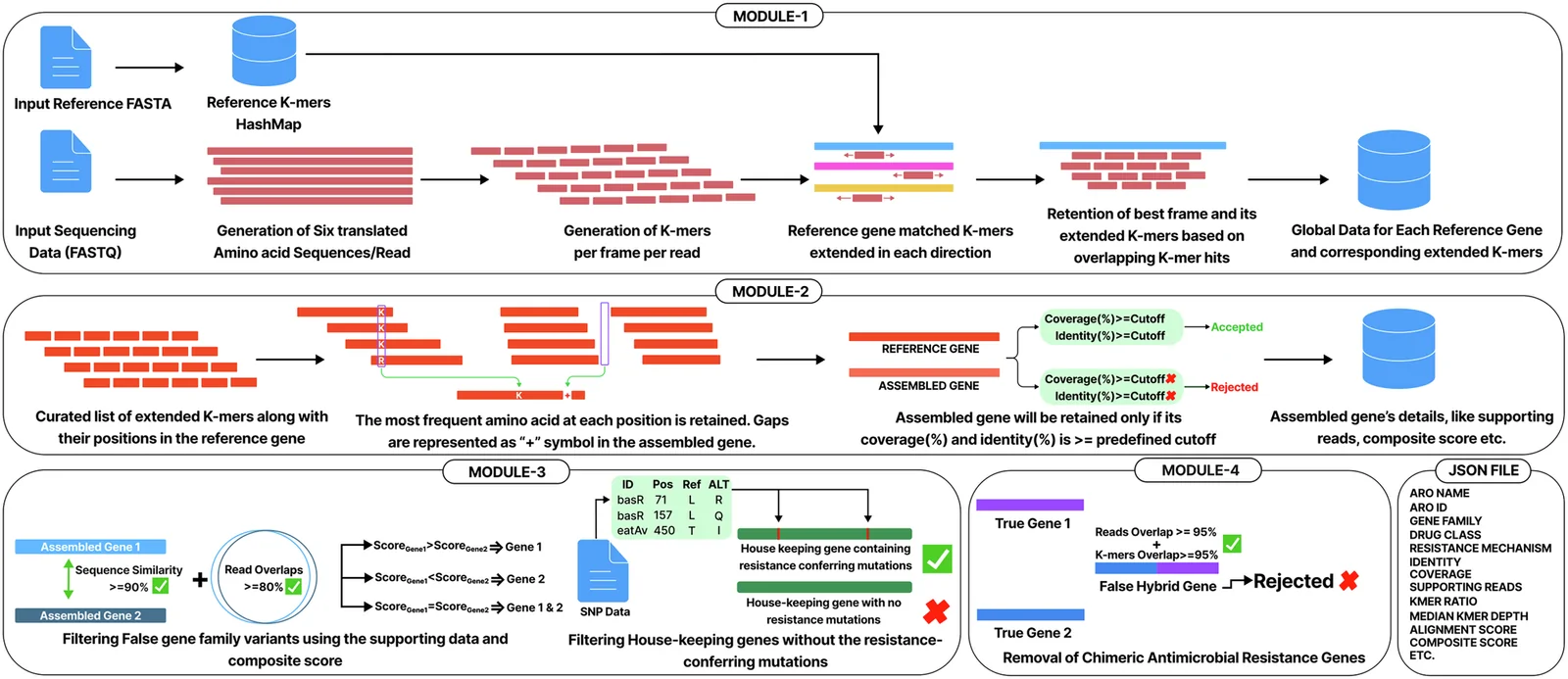
Schematic of the K-MARVEL Algorithm. K-MARVEL is divided into four modules to capture ARGs from raw sequencing data. (1) Read translation and k-mer generation, followed by best frame selection, (2) ARG assembly and filtering by coverage/identity cutoffs, (3) filtering of false gene family variants and house-keeping genes without the resistance-conferring mutations, and (4) removal of false chimeric ARGs. The final output provides detailed information about the assembled genes in JSON format. Figure was created using Figma (https://www.figma.com/).

K-MARVEL is a high-performance computational tool implemented in Rust for identifying ARGs from DNA sequencing data. The tool is optimized for both short-read and long-read sequencing technologies. The workflow consists of four modules, each described below. The schematic overview of the modules involved in K-MARVEL is presented in Fig. 4.

### Module 1: Reference database indexing and read mapping

The tool starts by indexing a reference database of known ARG protein sequences provided by the user. The protein sequences are indexed into a hash map with unique k-mers of size *k* as keys and sequence headers as values.

Given a reference amino acid sequence of length *n*,

$$r=\{({r}_{1},{r}_{2},\cdots,{r}_{n})|{r}_{i}\in \{{amino\; acid}\}\}$$

generate the set of possible k-mers of length $k\in {{\mathbb{Z}}}_{+}^{n},k\le n$ as

$$S(r,k)=\{r[i..i+k-1]{|i}\in \{1,2,\cdots,n-k+1\}\}$$

The FASTQ file provided by the user is processed in a streamlined fashion to maintain a low memory footprint. For each read, the DNA sequence is translated into all six translated frames (considering reverse complementarity). Given a DNA sequence of length *n*,

$$s=\{({s}_{1},{s}_{2},\cdots,{s}_{n})|{s}_{i}\in \{A,T,G,C\}\}$$

and its reverse complement

$${s}^{r}=\left(c\left({s}_{n}\right),c\left({s}_{n-1}\right),\cdots,c\left({s}_{1}\right)\right)$$

where $c({s}_{i})$ is the complementary nucleotide of ${s}_{i}$, for each frame $f\in \{{{\mathrm{0,1,2}}}\}$ naively translate each codon into its amino acid using the codon table $T:{\{A,T,G,C\}}^{3}\to \{{amino\; acid}\}$. Given *m* valid codons in frame *f*:

$$A(s,f)=\{T(s[i..i+2]),\cdots,T(s[i+3m-2..i+3m])\}$$

and

$$A({s}^{r},f)=\{T({s}^{r}[i..i+2]),\cdots,T({s}^{r}[i+3m-2..i+3m])\}$$

The six translated frames are converted into k-mers of size *k* as described. These k-mers are then matched with the reference database, and if any k-mers are perfectly matched with a reference k-mer, they are extended by an extension $e\in {{\mathbb{Z}}}_{+}^{n},e\le \frac{m-k+1}{2}$ on both ends. Given a matching k-mer

$${K}_{{match}}=A(s,f)[i..i+k-1]$$

the extended k-mer

$${K}_{{extended}}=A(s,f)[\max (1,i-e)..\min (m,i+k-1+e)]$$

To reduce spurious k-mer matches, a read is kept for further analysis only if, in at least one of its six reading frames, it contains a minimum of five overlapping extended k-mers that map to a reference gene. Information regarding the extended k-mers, including read ID, frame ID, and frame positions, is stored for further analysis.

### Module 2: Assembling ARGs

Since only one frame can encode the gene, only the best (read id, frame) pair is kept based on the number of overlapping matchings of extended k-mers. Given two extended k-mers, *K*_*1*_ and *K*_2_, matching a given reference gene sequence ***r*** of length *n*, their ranges are determined as the absolute positions in that sequence where they match. Let the matching range of ${K}_{1}$ on ***r*** be denoted as

$${Range}({K}_{1})=\{[{p}_{1}..{q}_{1}]|{p}_{1},{q}_{1}\in {{\mathbb{N}}}_{+}^{n},{p}_{1} > {q}_{1}\}$$

and the matching range of ${K}_{2}$ on ***r*** be denoted as

$${Range}({K}_{2})=\{[{p}_{2}..{q}_{2}]|{p}_{2},{q}_{2}\in {{\mathbb{N}}}_{+}^{n},{p}_{2} > {q}_{2}\}$$

Then ${K}_{1}$ and ${K}_{2}$ are considered overlapping if the following conditions are met.

$${p}_{1}\le {q}_{2}\,{{\mathrm{and}}}\,{p}_{2}\le {q}_{1}$$

and

The number of overlapping extended k-mers is counted for each frame, and the frame with the highest number of overlapping k-mers is retained for the downstream analysis. If none of the frames has at least five overlapping extended k-mers, the read is excluded from further analysis of the target resistance gene.

Once all extended k-mers are curated for the reference gene, they are utilized to calculate the gene coverage. Gene coverage is computed as the percentage of amino acids in the reference gene covered at least once by the matching k-mers. Given the total number of amino acids covered at least once as ${n}_{{covered}}$, the gene coverage is

$$100\cdot \frac{{n}_{{covered}}}{n}$$

The assembly step will be done only if the gene coverage is more than or equal to the pre-defined coverage threshold.

During the assembly step, a consensus protein sequence for the target gene is constructed from the retained extended k-mers. The extended k-mers are aligned to the reference gene based on their positional coordinates. At each reference gene position, the amino acids contributed by the overlapping extended k-mers are counted, and the most frequent amino acid is selected for the consensus sequence. If two or more amino acids share the highest frequency and one of them matches the reference amino acid, the reference amino acid is retained for that position. In the rare case where multiple non-reference amino acids are tied for the highest frequency, all tied amino acids are recorded and reported in the final output file. The reference gene location for which no amino acid can be curated due to the absence of reference k-mers is represented by the “+” symbol, i.e., gaps in the assembled sequence are represented by the “+” symbol. To be in concordance with the assembly-based ARG detection tools, the identity (%) is calculated by employing the Smith-Waterman based Local alignment with BLOSUM62 scoring metrics provided by the rust-bio crate version 2.3.0^33^. The assembled genes with an identity (%) greater than or equal to the pre-defined cutoff set by the user will be retained for further analysis.

To filter the false variants in the downstream analysis, a composite score is derived from (I) the sequence identity to the original reference gene sequence, employing the Smith-Waterman algorithm provided in the rust-bio crate version 2.3.0^33^; (II) k-mer ratio, defined as the ratio of reference k-mers captured in the data and the total number of possible reference k-mers n−k+1; and (III) normalised alignment score, defined as the alignment score calculated by the global alignment normalised by the reference gene length. The formula for the composite score is provided in Eq. 1.

1 $$\frac{1}{3}\cdot \left({Identity}+k-{merratio}+{NormalisedAlignmentScore}\right)$$

### Module 3: Filtering false variants and house-keeping genes

Because of the high sequence similarity between the members of the same gene family, there is a possibility of assembling false variants. These variants are filtered based on the two-step strategy.

Our initial assumption, that each read would encode only one gene, holds true for short-read sequencing data. However, long reads frequently span multiple adjacent genes, meaning the same set of reads may support multiple gene assemblies. To address this, the tool first performs a pairwise read overlap analysis between assembled genes. If the overlapping reads constitute ≥ 80% of the total reads supporting either gene in the pair, the tool proceeds to evaluate sequence similarity between the assembled sequences of the gene pair. It uses the algorithm to compute the longest common subsequence between two strings to measure sequence similarity. If the longest common subsequence is at least 90% of the smallest gene in the pair, the gene with the highest composite score will be retained. If both genes in the pair have the same composite score, then the tool will retain both.

Certain housekeeping genes/structural genes also confer antibiotic resistance only in the presence of specific mutations. To capture these mutated housekeeping genes, the tools perform a secondary screen against the curated set of resistance-causing mutations provided by the user. Genes containing at least one curated mutation from the known set of mutations will be retained for further analysis.

### Module 4: Removal of chimeric artifacts

In samples containing multiple variants of the same gene family, the tool may assemble a false chimeric gene by combining extended k-mers derived from distinct true variants. To remove such chimeric artifacts, the method applies a two-step filtering approach based on both extended k-mers and supporting read evidence. First, assembled ARGs are grouped by gene family, and the percentage of unique extended k-mers is calculated for each gene. For genes in which ≥95% of the supporting k-mers are shared with other members of the same gene family, the percentage of unique supporting reads is then calculated. Genes with both a unique extended k-mer percentage below 5% and a unique supporting read percentage below 5% are identified as chimeric artifacts and removed from the final set.

To ensure concordance with assembly-based ARG-calling tools, K-MARVEL performs a protein BLAST^34^ search against the final set of assembled ARGs. This step retrieves BLAST-based ARG classification along with other relevant metrics, such as E-value and Bit score. Protein BLAST is applied exclusively to genes classified under the protein homology model. For these genes, the final output reports both the native K-MARVEL annotation and the protein BLAST-based annotation, enabling users to select the preferred classification. For classification performance evaluation against other read-based methods, BLAST-based annotations were labeled K-MARVEL (BLAST), whereas native annotations were labeled K-MARVEL (NATIVE).

After running the final module, the tool will produce the resulting output as a JSON and tab-separated value (tsv) file. The main reason behind outputting the data in JSON format is to ease the tool’s integration with other pipelines. Each gene in the JSON data will include information on the assembled gene sequence, mutations found in the assembled gene, and other relevant metadata such as gene family, drug class, etc.

### Calculation of optimized K-mer and Extension size for Short and Long-read sequencing data

To determine the optimal k-mer and extension sizes for the sequencing data, we used 12 samples, each representing a unique species, for which short-read and long-read sequencing data and reference-level genome assemblies were available in the public repository. To establish the ground truth set of ARGs in these 12 reference assemblies, we first downloaded the CDS protein FASTA file from NCBI^35^ and performed protein BLAST^34^ searches using the CARD homology-model protein FASTA file as the reference database. For each query protein sequence, the top hit meeting 90% identity and coverage was selected as the ground truth for the homology model. For the overexpression and variant models, we used RGI version 6.0.5^16^ with the ‘--include-loose‘ parameter. ARGs that exhibited 90% identity and coverage, along with the resistance-conferring mutations, were subsequently selected as the ground truth for these models.

Once the samples’ ground truth set was finalized, the tool was executed with k-mer sizes ranging from 6 to 10 and extension sizes ranging from 6 to 12, with an identity (%) cutoff of 90 and a coverage (%) cutoff of 90. The True Positives (TP), False Positives (FP), and False Negatives (FN) were calculated for each pair of the k-mer and extension. These metrics were aggregated by the k-mer and extension size for all twelve samples, and the F1 score (Eq. 4), the harmonic mean of Precision (Eq. 2) and Recall (Eq. 3), was calculated based on the aggregated TP, FP, and FN. The k-mer extension pair with the highest F1-score was selected as the default k-mer and extension size for the sequencing technology.

2 $${Precision}=\frac{{TRUE\; POSITIVES}\left({TP}\right)}{{TRUE}\,{POSITIVES}\left({TP}\right)+{FALSE}\,{POSITIVES}\left({FP}\right)}$$

3 $${Recall}=\frac{{TRUE}\,{POSITIVES}\left({TP}\right)}{{TRUE}\,{POSITIVES}\left({TP}\right)+{FALSE}\,{NEGATIVES}\left({FN}\right)}$$

4 $$F1-{SCORE}=\frac{2\cdot {PRECISION}\cdot {RECALL}}{{PRECISION}+{RECALL}}$$

5 $${Accuracy}=\frac{{TP}+{TN}}{{TP}+{TN}+{FP}+{FN}}$$

### Systematic benchmarking of K-MARVEL with other read-based ARG classification tools

To evaluate the ARG classification accuracy of K-MARVEL, we benchmarked its performance against widely used read-based ARG classification tools on both short-read and long-read sequencing datasets. For short-read datasets, we selected ARGprofiler version 1.0.0^36^, DeepARG version 2.0.0^37^, KARGA version 1.02^38^, KmerResistance version 2.0.0^19,39^, RGI-BWT version 6.0.5^16^, ShortBRED version 0.9.3^40^ and SRST2 version 0.2.0^41^. For long-read datasets, ABRicate version 1.2.^42^ was selected because it is included in the metagenomics workflow from Oxford Nanopore Technologies (ONT). Within this workflow, ABRicate is used for ARG detection in both read-based and assembly-based routes, and it has been widely employed in previous studies for direct ARG identification from long read sequencing data^28,43,44^. KARGA version 1.02^38^, which supports both short- and long-read sequencing data, was also incorporated into the benchmarking. As these read-based classification tools do not support the detection of resistance-conferring mutations, benchmarking was limited to assessing their accuracy in capturing homology model-based ARGs.

Benchmarking was performed on short-read and long-read datasets comprising 205 and 209 samples, respectively, distributed across 22 bacterial species, the majority of which are included in the WHO priority pathogen list^18^. Sample distribution by species and sequencing technology is shown in the Supplementary Fig. 4 and Supplementary Data 10.

The ground truth set for each sample was curated using the same approach across the 12 datasets for k-mer and extension-size optimization. The CDS protein FASTA files were either obtained from NCBI or generated using the NCBI Prokaryotic Genome Annotation Pipeline (PGAP) build 7983^45^. For the homology model, protein BLAST is performed against the CARD homology-model protein FASTA database, and for each query sequence, the top hit with ≥ 90% coverage is selected as ground truth. For overexpression and variant models, RGI was executed with the ‘--include-loose‘ parameter, and ARGs meeting the 90% identity and coverage threshold and containing the resistance-conferring mutations are subsequently selected as ground truth.

### Statistics and reproducibility

All statistical analyses in this study were described in the text and performed using R version 4.4.1 (https://www.r-project.org/). Figures were generated using the following R packages: Tidyverse version 2.0.0^46^, ggpubr version 0.6.3 (https://rpkgs.datanovia.com/ggpubr/), cowplot version 1.2.0 (https://wilkelab.org/cowplot/), GGTREE version 3.14.0^47^, scales version 1.4.0 (https://scales.r-lib.org), rstatix version 0.7.3 (https://rpkgs.datanovia.com/rstatix/), patchwork version 1.3.2 (https://patchwork.data-imaginist.com/). All benchmarking tools used in the study are publicly available, with their versions indicated in the text.

The datasets were selected from published research and include sequencing data (both short-read and long-read) from 22 bacterial species, the majority of which are on the WHO priority pathogen list. In total, 205 short-read datasets and 209 long-read datasets were used to benchmark K-MARVEL’s performance. No statistical method was used to predetermine sample size, and no statistics were used to infer expected performance outside the tested datasets, rather, the tools were compared directly to highlight the characteristics of our method. No data were excluded from the analyses. The experiments were not randomized, and the investigators were not blinded to allocation during experiments and outcome assessment, as all comparisons were based on objective, automated computational benchmarks.

### Reporting summary

Further information on research design is available in the Nature Portfolio Reporting Summary linked to this article.

## Supplementary information

Supplementary Information

Description of Additional Supplementary Files

Supplementary Data 1

Supplementary Data 2

Supplementary Data 3

Supplementary Data 4

Supplementary Data 5

Supplementary Data 6

Supplementary Data 7

Supplementary Data 8

Supplementary Data 9

Supplementary Data 10

Reporting Summary

Transparent Peer Review file

## Source data

Source Data

## Data Availability

Source data are provided in this paper. No new data were generated during this study. All benchmarking sequencing datasets are publicly accessible in the NCBI Sequence Read Archive (SRA). Supplementary Data 10 contains a complete list of the 414 datasets with their metadata, including direct links to each dataset. Source data are provided with this paper.

## Code availability

K-MARVEL is implemented in Rust, is open source, and is freely available at https://bitbucket.org/amr-avengers/k-marvel/src/main/.
