# Supplementary material for: K-MARVEL: K-Mer-based antimicrobial resistance virtual exploration lab
Source: Nat Commun. 2026 Sep 3;17:10505. doi: 10.1038/s41467-026-77438-8 (PMC13639074; doi:10.1038/s41467-026-77438-8)
Supplement: Supplementary file 1 [file 41467_2026_77438_MOESM13_ESM.pdf]

nature portfolio

Corresponding author(s): Rafi Ahmad

Last updated by author(s): Jul 31, 2026

Reporting Summary

Nature Portfolio wishes to improve the reproducibility of the work that we publish. This form provides structure for consistency and transparency in reporting. For further information on Nature Portfolio policies, see our Editorial Policies and the Editorial Policy Checklist.

Statistics

For all statistical analyses, confirm that the following items are present in the figure legend, table legend, main text, or Methods section.

n/a	Confirmed	
☐	☒	The exact sample size (*n*) for each experimental group/condition, given as a discrete number and unit of measurement
☐	☒	A statement on whether measurements were taken from distinct samples or whether the same sample was measured repeatedly
☐	☒	The statistical test(s) used AND whether they are one- or two-sided<br>*Only common tests should be described solely by name; describe more complex techniques in the Methods section.*
☒	☐	A description of all covariates tested
☐	☒	A description of any assumptions or corrections, such as tests of normality and adjustment for multiple comparisons
☐	☒	A full description of the statistical parameters including central tendency (e.g. means) or other basic estimates (e.g. regression coefficient) AND variation (e.g. standard deviation) or associated estimates of uncertainty (e.g. confidence intervals)
☐	☒	For null hypothesis testing, the test statistic (e.g. F, t, r) with confidence intervals, effect sizes, degrees of freedom and P value noted<br>*Give P values as exact values whenever suitable.*
☒	☐	For Bayesian analysis, information on the choice of priors and Markov chain Monte Carlo settings
☒	☐	For hierarchical and complex designs, identification of the appropriate level for tests and full reporting of outcomes
☐	☒	Estimates of effect sizes (e.g. Cohen's *d*, Pearson's *r*), indicating how they were calculated

Our web collection on statistics for biologists contains articles on many of the points above.

Software and code

Policy information about availability of computer code

Data collection	No new data has been generated during this study. All sequencing datasets used for benchmarking are publicly available in the NCBI Sequence Read Archive (SRA). A complete list of all 414 datasets along with their corresponding metadata is provided in Supplementary Data 10, which also includes direct hyperlinks to each dataset.
Data analysis	Data analysis was performed using the following tools:<br>ARGprofiler version 1.0.036<br>DeepARG version 2.0.037<br>KARGA version 1.0238<br>KmerResistance version 2.0.019,39<br>RGI-BWT version 6.0.516<br>ShortBRED version 0.9.340<br>SRST2 version 0.2.041<br>ABRicate version 1.2.0<br>K-MARVEL, source code is available from https://bitbucket.org/amr-avengers/k-marvel/src/main/<br><br>All plots were made using R version 4.4.1 using the following packages: tidyverse version 2.0.0, ggpubr version 0.6.3, cowplot version 1.2.0, ggtree version 3.14.0, scales version 1.4.0, rstatix version 0.7.3, patchwork version 1.3.2.

For manuscripts utilizing custom algorithms or software that are central to the research but not yet described in published literature, software must be made available to editors and reviewers. We strongly encourage code deposition in a community repository (e.g. GitHub). See the Nature Portfolio guidelines for submitting code & software for further information.

Data

Policy information about availability of data

All manuscripts must include a data availability statement. This statement should provide the following information, where applicable:

- Accession codes, unique identifiers, or web links for publicly available datasets
- A description of any restrictions on data availability
- For clinical datasets or third party data, please ensure that the statement adheres to our policy

K-MARVEL is open-source software distributed under the LGPL and is available from https://bitbucket.org/amr-avengers/k-marvel/src/main/

Research involving human participants, their data, or biological material

Policy information about studies with human participants or human data. See also policy information about sex, gender (identity/presentation), and sexual orientation and race, ethnicity and racism.

Reporting on sex and gender	NA
Reporting on race, ethnicity, or other socially relevant groupings	NA
Population characteristics	NA
Recruitment	NA
Ethics oversight	NA

Note that full information on the approval of the study protocol must also be provided in the manuscript.

Field-specific reporting

Please select the one below that is the best fit for your research. If you are not sure, read the appropriate sections before making your selection.

☒ Life sciences ☐ Behavioural & social sciences ☐ Ecological, evolutionary & environmental sciences

For a reference copy of the document with all sections, see nature.com/documents/nr-reporting-summary-flat.pdf

Life sciences study design

All studies must disclose on these points even when the disclosure is negative.

Sample size	No statistical method was used to predetermine sample size and all the used samples were part of the publicly available data sets. The data sets were chosen to contain sequencing data (both short and long read sequencing) from 22 bacterial species, the majority of which are included in the WHO priority pathogen list. 209 datasets from long-read sequencing and 205 datasets from short-read sequencing was used to test the performance of K-MARVEL.<br><br>No statistics were used to infer how performance may be expected for sequencing data outside of the tested data sets. Rather the tools were directly compared to highlight the characteristics of our method.
Data exclusions	No dataset was excluded from the analysis.
Replication	All data and software used are publicly available.
Randomization	There was no group allocation.
Blinding	There was no group allocation.

Reporting for specific materials, systems and methods

We require information from authors about some types of materials, experimental systems and methods used in many studies. Here, indicate whether each material, system or method listed is relevant to your study. If you are not sure if a list item applies to your research, read the appropriate section before selecting a response.

Materials & experimental systems

n/a	Involved in the study
☒	☐ Antibodies
☒	☐ Eukaryotic cell lines
☒	☐ Palaeontology and archaeology
☒	☐ Animals and other organisms
☒	☐ Clinical data
☒	☐ Dual use research of concern
☒	☐ Plants

Methods

n/a	Involved in the study
☒	☐ ChIP-seq
☒	☐ Flow cytometry
☒	☐ MRI-based neuroimaging

Plants

Seed stocks	NA
Novel plant genotypes	NA
Authentication	NA
