# Supplementary material for: K-MARVEL: K-Mer-based antimicrobial resistance virtual exploration lab
Source: Nat Commun. 2026 Sep 3;17:10505. doi: 10.1038/s41467-026-77438-8 (PMC13639074; doi:10.1038/s41467-026-77438-8)
Supplement: Supplementary file 2 [file 41467_2026_77438_MOESM14_ESM.pdf]

nature portfolio

Peer Review File

K-MARVEL: K-Mer-based Antimicrobial Resistance Virtual Exploration Lab

Corresponding Author: Professor Rafi Ahmad

This file contains all reviewer reports in order by version, followed by all author rebuttals in order by version.

Version 0:

Reviewer comments:

Reviewer #1

(Remarks to the Author)
General comment.

In this study, the authors present a new tool to detect AMR-conferring genes and mutations directly from raw reads, either sequenced by Illumina or ONT. The paper is clearly written and the language is appropriate throughout. I have to commend the lengths at which the authors went to describe and benchmark their tool, especially on handling large-scale sequencing datasets. I have few comments, mainly related to the methodology used by the authors, which I list hereafter, sorted from major to minor.

Major comments.

L82-84: The inclusion of the 12 pathogens to determine the optimal k-mer and extension sizes is lacking explanations which are not covered by the methods section. Could the authors expand on
1) the metrics related to these specific samples and why were they selected (e.g., overall read quality, the ONT sequencing chemistry used, which could impact the detection…);
2) Why exactly 12? The authors mention that 22 bacterial species were used in the benchmarking against other tools, potentially more species could be used to fine tune the tool as well.

L122: Other tools exist to detect AMR genes (e.g., ShortBRED, DeepARG, KmerResistance), which could potentially work in the benchmarking scenario presented here. Could the authors comment on if these tools were considered and why were they not retained?

L533: From the documentation, ABRicate mentions requiring contigs (or very long high quality reads). How did the authors ensure that this condition was met? Alternatively, the comparison to actual read-based gene detection tools will unfairly bias the results from ABRicate.

Minor comments.

L54: Could the authors also put in perspective the comparison of Illumina to the new ONT R10.4 chemistry? I feel it is a missing point in the introduction, as the new chemistry enables the generation of long reads close in quality to short reads, suggesting improvement in assembly quality, bypassing the need for hybrid sequencing, and the detection of ARGs and point mutations (see e.g., Hall et al. 2025: https://doi.org/10.7554/eLife.98300.3.sa0 or Sanderson et al. 2024: https://doi.org/10.1099/mgen.0.001246)

L82: For the sake of reproducibility, could the authors provide accession numbers as well, e.g., in supplementary table s1?

L103, 381: ARG is already defined in the introduction.

L122,183: KMARVEL -> K-MARVEL

L131: Since F1-score is used as the main metric, could the authors comment on the decision of including accuracy in the

figure?

L412 and L427: Could the authors explain why the threshold of five is used and how it was set? How does read length influence this filtering?

L442: The authors seem to use frequency, depth and occurrence of counts (L439) interchangeably. Could the authors clarify this section?

L464: It's unclear how neighboring genes and "gene pair" are connected in this context. Is the tool looking for pairs of genes on a single read? Could the authors rephrase for clarity?

Supplementary material: Could the authors add, in the supplementary document, legends corresponding to the supplementary tables?

(Remarks on code availability)
Comments on the code/repository:

Repository: small typos are present (KARMA instead of K-MARVEL, kmarvel instead of k-marvel in some commands) in the documentation.

Repository: "bash test_installation.sh" on linux (ubuntu 22.04) throws a "test_installation.sh: line 69: syntax error: unexpected end of file"

Repository: Running kmarvel using the provided test data yielded an error mentioning the absence of index file for the database. Could the authors expand their "usage" section in the documentation by explicitly mentioning that the CARD database should be indexed?

Repository: While a JSON file is powerful to store multiple information related to the detection of such ARGs and AMR-conferring mutations, would the authors consider adding a tabular-separated output, as it is a frequently used format facilitating downstream processing?

Reviewer #2

(Remarks to the Author)
This describes a method, K-MARVEL, for detecting antibiotic resistance genes in metagenomic data. This is an interesting method, which basically does 6-frame translation and then looking for AMR genes in amino-space. The method is reasonably clearly explained, and well motivated, and I enjoyed reading it, but I think the paper needs work to better explain itself.

Major questions:

1. Figure 1 is a horrible way to visualise and look at results; if we have to keep it, I suggest keep it in the supplement. I do not argue about using the grid search to find a good choice of parameter

2. I don't see where you explain what "variant and overexpression models" are?

3. Figure 2 - I don't think the phylogeny does anything useful here? You could drop it and give more space for panels B and C

4. KMARVEL (BLAST) is mentioned for the first time on line 145 without ever introducing what it is. Same with NATIVE

5. Table 3 - I think it would be good to make it clearer in more detail what the short and long read datasets are - eg in the table I'd suggest putting nanopore or pacbio and the year of sequencing (as the data changes a lot over time). I'd like to get a sense of how good the truth data is (the methods just says "The ground truth set for each sample was curated using the same approach across the 12 datasets for k-mer and extension-size optimization.". How is the truth assembly done, how do you know how good it is, how do you know what genes really are in it? What are the read length distributions like? What's the data quality like - is it 2017 , 2020, 2023 or 2025 nanopore data? These are all v different. How do you compare the calls of each tool with the truth?

6. Can you at the start of the results, explain how your method works - fine to push details into methods, but the reader wants to know what this is all about, and why this method is new. If you want to show kmer detection at the protein level is better - why not compare with kmer detection at the nucleotide level? If your answer is that tool XXXX in your benchmark does this, then go ahead and say so explicitly, so you can compare nucleotide versus protein/amino detection.

Minor issues

1. I do not think Module 4 (removal chimeric genes) is going to be very robust; the problem is that when you have many

many alleles of a single gene, some of them are likely to end up being recombinants, and so there will (in general) not be SNPs unique to alleles. Alleles will have unique combinations, but not unique SNPs or kmers. So, for some genes, I don't think the approach of penalizing low uniqueness makes sense.

(Remarks on code availability)

Version 1:

Reviewer comments:

Reviewer #1

(Remarks to the Author)
General comment

I would like to thank the authors for addressing all my comments thoroughly. I have only slighly minor comments about Figure 2.

Minor comments.

- Figure 2 labels are barely readable.
- Figure 2: some of the colors don't show in boxes of some subpanels (e.g., subpanel B). I would consider adding a legend in the x-axis labelling the tool corresponding to each box.

(Remarks on code availability)
The authors have addressed all my comments.

Open Access This Peer Review File is licensed under a Creative Commons Attribution 4.0 International License, which permits use, sharing, adaptation, distribution and reproduction in any medium or format, as long as you give appropriate credit to the original author(s) and the source, provide a link to the Creative Commons license, and indicate if changes were made.
In cases where reviewers are anonymous, credit should be given to 'Anonymous Referee' and the source.
The images or other third party material in this Peer Review File are included in the article's Creative Commons license, unless indicated otherwise in a credit line to the material. If material is not included in the article's Creative Commons license and your intended use is not permitted by statutory regulation or exceeds the permitted use, you will need to obtain permission directly from the copyright holder.
To view a copy of this license, visit https://creativecommons.org/licenses/by/4.0/

Reviewer #1 (Remarks to the Author):

General comment.

In this study, the authors present a new tool to detect AMR-conferring genes and mutations directly from raw reads, either sequenced by Illumina or ONT. The paper is clearly written, and the language is appropriate throughout. I have to commend the lengths to which the authors went to describe and benchmark their tool, especially on handling large-scale sequencing datasets. I have a few comments, mainly related to the methodology used by the authors, which I list hereafter, sorted from major to minor.

We thank the reviewers for their valuable comments to improve the quality of the manuscript. Below is the point-by-point response to the comments raised by the reviewers:

Major comments.

1. L82-84: The inclusion of the 12 pathogens to determine the optimal k-mer and extension sizes lacks explanations that are not covered by the methods section. Could the authors expand on:

A) the metrics related to these specific samples and why they were selected (e.g., overall read quality, the ONT sequencing chemistry used, which could impact the detection…);

Response: We thank the reviewer for their input. Based on your suggestion, we have added details on the sequencing data used for k-mer and extension-size optimization below (also in Supplementary Table 10). These samples were selected because they represent publicly available ONT datasets generated using the R10.4.1 chemistry (new chemistry, as the old chemistry is phased out), while also covering a broad range of read-length and read-quality profiles. Specifically, the datasets had read N50 values ranging from 5,289 bp to 14,491 bp and median Q-scores ranging from 28.49 to 39.53. This allowed us to evaluate whether the selected k-mer and extension parameters performed consistently across samples with both moderate and high read lengths, as well as across different levels of ONT read accuracy.

SRA ID	Chemistry	N50 Length (bp)	Median Q-Score	Submission Date
SRR24833096	R10.4.1	12,423	28.6335	06-06-2023
SRR24833073	R10.4.1	5,289	28.709	06-06-2023
SRR24833101	R10.4.1	6,777	30.2834	06-06-2023
SRR24833098	R10.4.1	10,985	28.4982	06-06-2023
SRR32486094	R10.4.1	12,382	38.1645	25-02-2025
SRR32486200	R10.4.1	10,867	37.7884	25-02-2025
SRR32486273	R10.4.1	8,666	39.5328	25-02-2025
SRR24833086	R10.4.1	14,491	28.7889	06-06-2023
SRR32486258	R10.4.1	8,309	38.602	25-02-2025
SRR24833080	R10.4.1	7,147	30.3903	06-06-2023
SRR24833090	R10.4.1	10,104	30.3492	06-06-2023
SRR32486158	R10.4.1	9,203	37.4647	25-02-2025

B) Why exactly 12? The authors mention that 22 bacterial species were used in the benchmarking against other tools; potentially, more species could be used to fine-tune the tool as well.

Response: We thank the reviewer for raising this important point. For k-mer and extension-size optimization, we selected 12 representative ONT samples, each corresponding to a distinct bacterial species, from the broader benchmarking dataset. These 12 samples were chosen to capture diversity in bacterial genome backgrounds while also covering a broad range of sequencing characteristics, including read length and read quality.

The purpose of using these 12 samples was not to optimize the tool for specific pathogen species, but to identify a robust k-mer and extension-size combination that performs consistently across different read-quality and read-length distributions. We expect these parameters to be influenced primarily by sequencing-related properties, such as read accuracy, error profile, and read-length distribution, rather than by species identity alone. After the optimal values were determined, K-MARVEL was run using the fixed parameter set on the complete benchmarking dataset, including all 22 bacterial species. Hence, our optimization and benchmarking are agnostic to species-specific or sample-specific parameters.

The selected 12-sample optimisation set provided a practical and diverse subset for systematic parameter evaluation. Also, the full 22-species dataset was used to assess the final performance of the tool against other methods.

2. L122: Other tools exist to detect AMR genes (e.g., ShortBRED, DeepARG, KmerResistance), which could potentially work in the benchmarking scenario presented here. Could the authors comment on whether these tools were considered and why they were not retained?

Response: We thank the reviewer for their insightful comment. Based on their suggestion, we have incorporated ShortBRED, DeepARG and KmerResistance in the benchmarking for short-read sequencing datasets. The analysis revealed that K-MARVEL outperformed all the other seven tools with an F1-score of 0.979. For your reference, we are adding the table containing global classification metrics for all short-read-based classification methods.

**Method**	**True Positive (TP)**	**False Positive (FP)**	**False Negative (FN)**	**Precision**	**Recall**	**F1-Score**	**Accuracy**
ARGProfiler	4532	334	762	0.931	0.856	0.892	0.805
DeepARG	2925	3271	2369	0.472	0.552	0.510	0.341
KARGA	1438	2191	3856	0.396	0.272	0.322	0.192
**K-MARVEL (BLAST)**	**5208**	**140**	**86**	**0.974**	**0.984**	**0.979**	**0.958**
K-MARVEL (NATIVE)	5183	167	111	0.969	0.979	0.974	0.949
KmerResistance	4529	331	765	0.932	0.855	0.892	0.805
RGI-BWT	4641	434	653	0.914	0.876	0.895	0.810
ShortBRED	4282	1211	1012	0.779	0.810	0.794	0.658
SRST2	4455	698	839	0.864	0.842	0.853	0.744

3. L533: From the documentation, ABRicate mentions requiring contigs (or very long, high-quality reads). How did the authors ensure that this condition was met? Alternatively, the comparison to actual read-based gene detection tools will unfairly bias the results from ABRicate.

Response: We thank the reviewer for their comment. The main reason behind incorporating ABRicate in the benchmark is its inclusion in the EPI2ME workflow provided by the ONT team. In the EPI2ME workflow, both read-based and assembly-based routes utilize ABRicate for capturing ARGs. Because of this, we have also added ABRicate for the benchmarking analysis. Additionally, several studies have also utilized ABRicate for capturing ARGs directly from long-read sequencing data[1–3]. The information has also been added to the updated manuscript.

Page 22: L537-541:

"For long-read datasets, ABRicate version 1.2.0[42] was selected because it is included in the metagenomics workflow released by Oxford Nanopore Technologies (ONT). Within this workflow, ABRicate is used for ARG detection in both read-based and assembly-based routes, and it is widely employed in previous studies for direct ARG identification from long-read sequencing data[28,43,44]."

Minor comments.

1. L54: Could the authors also put in perspective the comparison of Illumina to the new ONT R10.4 chemistry? I feel it is a missing point in the introduction, as the new chemistry enables the generation of long reads close in quality to short reads, suggesting improvement in assembly quality, bypassing the need for hybrid sequencing, and the detection of ARGs and point mutations (see e.g., Hall et al. 2025: https://doi.org/10.7554/eLife.98300.3.sa0 or Sanderson et al. 2024: https://doi.org/10.1099/mgen.0.001246)

Response: We thank the reviewer for the comment. Based on your suggestion, we have added this information to the introduction section of the updated manuscript.

Page 3: L57-60:

"However, the new R10.4.1 chemistry enables the generation of high-quality long reads that approach short-read accuracy, potentially improving assembly accuracy, bypassing the need for hybrid sequencing and enhancing the detection of resistance genes and resistance-conferring mutations[6,7]."

2. L82: For the sake of reproducibility, could the authors provide accession numbers as well, e.g., in supplementary table S1?

Response: Thank you for your suggestion. We have added the supporting metadata in the supplementary table 10, including the assembly and SRA accession numbers.

3. L103, 381: ARG is already defined in the introduction.

Response: Thank you for pointing it out. The suggested changes are incorporated in the updated manuscript.

4. L122,183: KMARVEL -> K-MARVEL

Response: We thank the reviewers for catching this error in the manuscript. The suggested changes are incorporated in the updated manuscript.

5. L131: Since the F1-score is used as the main metric, could the authors comment on the decision of including accuracy in the figure?

Response: We thank the reviewer for this comment. To clarify, we included accuracy in the figure for two reasons. First, accuracy is a widely recognised and intuitive metric that is more familiar to clinicians and wet-lab researchers who may not be as accustomed to F1-score. Second, presenting both metrics allows readers to assess performance from multiple perspectives: F1-score for balanced evaluation and accuracy for overall correctness.

6. L412 and L427: Could the authors explain why the threshold of five is used and how it was set? How does read length influence this filtering?

Response: We thank the reviewer for their valuable feedback. In K-MARVEL, a read is considered for a reference gene only if at least one of its six reading frames contains a minimum of five overlapping k-mers specific to that gene. This threshold, determined through internal benchmarking during development, is independent of read length, as it serves solely as a pre-screening step to exclude reads that do not encode the reference gene. For reads passing this filter, the reading frame with the highest number of overlapping k-mers is selected as the representative frame for that read-gene pair and used for subsequent analysis.

This information is also provided in the updated manuscript.

Page 18: L428-431:

"The number of overlapping extended k-mers is counted for each frame, and the frame with the highest number of overlapping k-mers is retained for the downstream analysis. If none of the frames has at least five overlapping extended k-mers, the read is excluded from further analysis of the target resistance gene."

7. L442: The authors seem to use frequency, depth and occurrence of counts (L439) interchangeably. Could the authors clarify this section?

Response: We thank the reviewer for the feedback. Based on the suggestion, we have rewritten the paragraph with clarity in the updated manuscript.

Page 19: L439-446:

"During the assembly step, a consensus protein sequence for the target gene is constructed from the retained extended k-mers. The extended k-mers are aligned to the reference gene based on their positional coordinates. At each reference gene position, the amino acids contributed by the overlapping extended k-mers are counted, and the most frequent amino acid is selected for the consensus sequence. If two or more amino acids share the highest frequency and one of them matches the reference amino acid, the reference amino acid is retained for that position. In the rare case where multiple non-reference amino acids are tied for the highest frequency, all tied amino acids are recorded and reported in the final output file."

8. L464: It's unclear how neighbouring genes and "gene pair" are connected in this context. Is the tool looking for pairs of genes on a single read? Could the authors rephrase for clarity?

Response: We thank the reviewer for the feedback. Based on the suggestion, we have rewritten the paragraph with clarity in the updated manuscript.

Page 19,20: L464-473:

"Our initial assumption, that each read would encode only one gene, holds true for short-read sequencing data. However, long reads frequently span multiple adjacent genes, meaning the same set of reads may support multiple gene assemblies. To address this, the tool first performs a pairwise read overlap analysis between assembled genes. If the overlapping reads constitute $\geq$ 80% of the total reads supporting either gene in the pair, the tool proceeds to evaluate sequence similarity between the assembled sequences of the gene pair. It uses the algorithm to compute the longest common subsequence between two strings to measure sequence similarity.

If the longest common subsequence is at least 90% of the smallest gene in the pair, the gene with the highest composite score will be retained. If both genes in the pair have the same composite score, then the tool will retain both."

9. Supplementary material: Could the authors add, in the supplementary document, legends corresponding to the supplementary tables?

Response: We thank the reviewer for this valuable comment. Based on the suggestion, we have added the legends corresponding to each supplementary table in the supplementary figures file.

Reviewer #1 (Remarks on code availability):

Comments on the code/repository:

1. Repository: small typos are present (KARMA instead of K-MARVEL, kmarvel instead of k-marvel in some commands) in the documentation.

Response: We thank the reviewer for pointing out this error. We have fixed the typos in the README file.

2. Repository: "bash test_installation.sh" on Linux (Ubuntu 22.04) throws a "test_installation.sh: line 69: syntax error: unexpected end of file"

Response: We thank the reviewer for testing our tool. We have fixed the issue in the updated version of the code.

3. Repository: Running k-marvel using the provided test data yielded an error mentioning the absence of an index file for the database. Could the authors expand their "usage" section in the documentation by explicitly mentioning that the CARD database should be indexed?

Response: We thank the reviewer for pointing out the valuable error in the script. The setup script was missing the protein BLAST index step; we have added that in the updated version of the script and also added the information in the README file.

4. Repository: While a JSON file is powerful to store multiple information related to the detection of such ARGs and AMR-conferring mutations, would the authors consider adding a tabular-separated output, as it is a frequently used format facilitating downstream processing?

Response: We thank the reviewer for this valuable comment. Based on their suggestion, we have incorporated the changes in the updated version. The tool will output both JSON and TSV files at the end of the run, and the users can choose the output file based on their preference.

Reviewer #2 (Remarks to the Author):

This describes a method, K-MARVEL, for detecting antibiotic resistance genes in metagenomic data. This is an interesting method, which basically does 6-frame translation and then looks for AMR genes in amino-space. The method is reasonably clearly explained, and well-motivated, and I enjoyed reading it, but I think the paper needs work to better explain itself.

Major questions:

1. Figure 1 is a horrible way to visualise and look at results; if we have to keep it, I suggest keeping it in the supplement. I do not argue about using the grid search to find a good choice of parameters.

Response: We thank the reviewer for their valuable comment. Based on their suggestion, we have moved the figure to the Supplementary Data.

2. I don't see where you explain what "variant and overexpression models" are.

Response: We thank the reviewer for this comment. We have added the definitions of variant and overexpression models in the updated manuscript.

Page 6: L135-138:

"The protein variant model comprises housekeeping genes or antibiotic targets that confer resistance in the presence of specific point mutations. In contrast, the protein overexpression model encompasses regulatory proteins where specific mutations lead to increased copy number or upregulated expression, resulting in clinical resistance."

3. Figure 2 - I don't think the phylogeny does anything useful here? You could drop it and give more space for panels B and C.

Response: We thank the reviewer for their valuable feedback. Based on their suggestion, we have moved the phylogenetic tree to the Supplementary Data. Please find the modified Figure 2 attached below:

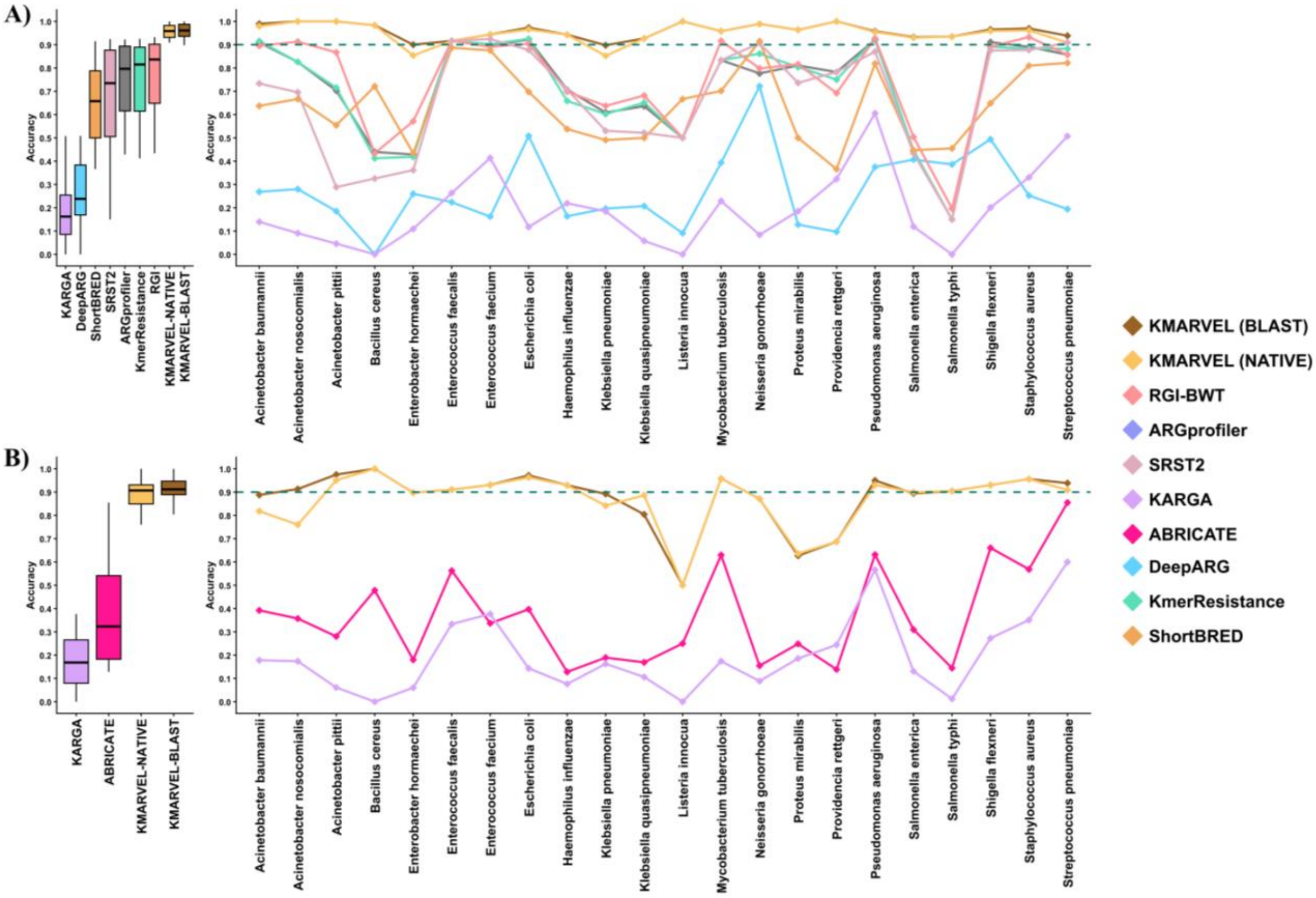

4. KMARVEL (BLAST) is mentioned for the first time on line 145 without ever introducing what it is. Same with NATIVE.

Response: We thank the reviewer for this comment. We have added the definitions of K-MARVEL (BLAST) and K-MARVEL (NATIVE) in the updated manuscript.

Page 8: L166-168:

"K-MARVEL (NATIVE) represented the ARGs captured by K-MARVEL natively, whereas K-MARVEL (BLAST) represented the captured ARGs re-annotated using protein BLAST."

Page 20, 21: L494-496:

“For classification performance evaluation against other read-based methods, BLAST-based annotations were labeled K-MARVEL (BLAST), whereas native annotations were labeled K-MARVEL (NATIVE).”

5. A) Table 3 - I think it would be good to make it clearer in more detail what the short and long read datasets are - e.g., in the table, I'd suggest putting nanopore or PacBio and the year of sequencing (as the data changes a lot over time).

Response: We thank the reviewer for this comment. Based on your suggestion, we have added the information, along with the median q-score, N50 read length, and mean read length, to Supplementary Table 10 for all sequencing datasets used in this study.

B) I'd like to get a sense of how good the truth data is (the methods just say, "The ground truth set for each sample was curated using the same approach across the 12 datasets for k-mer and extension-size optimization.". How is the truth assembly done? How do you know how good it is? How do you know what genes are in it?

Response: We thank the reviewers for this comment. We restricted our analysis to samples with NCBI reference-level assemblies, the highest quality designation assigned by NCBI, and therefore did not perform de novo genome assembly.

To curate the ground truth for each sample, we used the Comprehensive Antibiotic Resistance Database (CARD) database in conjunction with protein BLAST and Resistance Gene Identifier (RGI). For the homology model, we ran protein BLAST against the CARD homology-model protein FASTA file using the reference genome’s CDS FASTA file as the query and selected the top hit with ≥ 90% identity and coverage. For the variant and overexpression models, we used RGI to identify ARGs and retained those meeting the same identity and coverage threshold while also carrying the corresponding resistance-conferring mutations. This curation strategy was applied consistently for both k-mer and extension-size optimization and for benchmarking K-MARVEL against other read-based classifiers.

We have also added this information in detail to the updated manuscript.

Page 23: L550-557:

“The ground truth set for each sample was curated using the same approach across the 12 datasets for k-mer and extension-size optimization. The CDS protein FASTA files were either obtained from NCBI or generated using the NCBI Prokaryotic Genome Annotation Pipeline

(PGAP) build 7983[8]. For the homology model, protein BLAST is performed against the CARD homology-model protein FASTA database, and for each query sequence, the top hit with ≥ 90% and coverage is selected as ground truth. For overexpression and variant models, RGI was executed with the `--include-loose` parameter, and ARGs meeting the 90% identity and coverage threshold and containing the resistance-conferring mutations are subsequently selected as ground truth."

C) What are the read length distributions like? What's the data quality like - is it 2017, 2020, 2023 or 2025 nanopore data? These are all very different.

Response: We thank the reviewer for their valuable feedback. For the reviewer's reference, we have included the distribution of median Q-scores and-read length's N50 for the long-read sequencing datasets used in this study. As shown in the distribution, the majority of the data are Nanopore reads from 2023–2025 (*2 were sequenced using older R9.4.1 chemistry, 20 were sequenced using PacBio sequencing, and the remaining 187 were sequenced using R10.4.1 chemistry*), with Q-scores reaching 20 and above. Additionally, as indicated in Supplementary Table 10, most datasets were submitted after 2022. Regarding the read length distribution, the histogram shows that the majority of reads' N50 fall within the expected range of ≤ 10 Kbp.

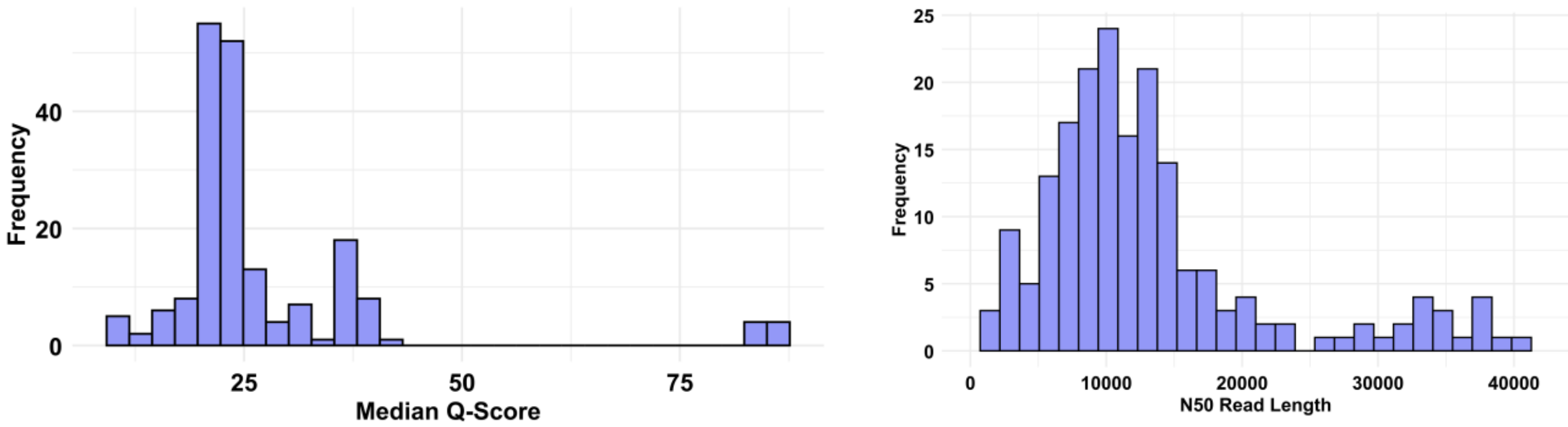

D) How do you compare the calls of each tool with the truth?

Response: We thank the reviewer for their valuable comment. To benchmark the classification performance of K-MARVEL and the other tested tools, we compared the ARO IDs of the captured genes against those of the ground truth for each dataset. As mentioned previously, the ground truth ARGs were curated from the RefSeq assemblies of the datasets. Since all tools were run against the CARD database, the ARO ID, a unique identifier for each gene, was retained in all outputs. We classified an ARO ID as a true positive if it was present in both the ground truth and the captured set, as a false positive if it appeared only in the captured set, and as a false negative if it appeared only in the ground truth.

6. Can you, at the start of the results, explain how your method works - fine to push details into methods, but the reader wants to know what this is all about, and why this method is new. If you want to show that kmer detection at the protein level is better, why not compare with kmer detection at the nucleotide level? If your answer is that tool XXXX in your benchmark does this, then go ahead and say so explicitly, so you can compare nucleotide versus protein/amino detection.

Response: We thank the reviewer for this valuable comment. Based on their suggestion, we have added the details about our method and other relevant information at the start of the results section.

Page 3,4: L82-89:

"We developed a novel method, named K-MARVEL, to capture ARGs directly from raw sequencing data from both short-read and long-read datasets. This method employs a k-mer match-and-extend strategy in protein space, enabling it to capture both homologous ARGs and genes carrying resistance-conferring mutations. K-MARVEL was further evaluated against other widely used read-based classifiers that either rely on read alignment or nucleotide k-mer-based detection. K-MARVEL consistently outperformed all evaluated tools in classification accuracy for both short-read and long-read datasets, demonstrating that protein-level detection substantially outperforms nucleotide-level approaches."

Minor issues

1. I do not think Module 4 (removal of chimeric genes) is going to be very robust; the problem is that when you have many, many alleles of a single gene, some of them are likely to end up being recombinants, and so there will (in general) not be SNPs unique to alleles. Alleles will have unique combinations, but not unique SNPs or kmers. So, for some genes, I don't think the approach of penalizing low uniqueness makes sense.

Response: We thank the reviewer for this valuable comment, and we generally agree. We did identify chimeras as an issue, though; hence, this module was designed to minimize false positives arising from chimeric assemblies that may be artificially constructed from k-mers originating from two or more highly similar ARGs within the same gene family. To distinguish genuine genes from such recombinants, the module leverages both supporting

reads and extended k-mers. If a candidate gene is chimeric, its supporting reads and k-mers will be shared across multiple true genes in the family, resulting in its exclusion from the final set. Conversely, even when k-mers or SNPs are not unique to a single gene, the supporting reads are generally unique to the true gene, ensuring that genuine genes are retained.

A potential limitation of this approach arises when two highly similar ARGs from the same family are adjacent on the genome and are spanned by long reads. In this scenario, the unique k-mers and supporting reads may fall below the threshold required to distinguish between the two genes, potentially leading to the erroneous removal of one of them. Notably, this read-spanning scenario is primarily relevant to long-read sequencing. In short reads, reads are generally shorter than full ARGs and are less likely to span two adjacent genes; therefore, adjacent genes are most likely to retain separable read-level support, provided that coverage exists over the regions that distinguish them. In such cases, assembly-based methods may outperform K-MARVEL, as they benefit from a longer genomic context to segregate these genes. However, based on our internal benchmarking, the likelihood of this scenario occurring is quite low.

Additionally, we have rewritten this part in the updated manuscript for more clarity.

Page 20: L480-488:

"In samples containing multiple variants of the same gene family, the tool may assemble a false chimeric gene by combining extended k-mers derived from distinct true variants. To remove such chimeric artefacts, the method applies a two-step filtering approach based on both extended k-mers and supporting read evidence. First, assembled ARGs are grouped by gene family, and the percentage of unique extended k-mers is calculated for each gene. For genes in which ≥95% of the supporting k-mers are shared with other members of the same gene family, the percentage of unique supporting reads is then calculated. Genes with both a unique extended k-mer percentage below 5% and a unique supporting read percentage below 5% are identified as chimeric artifacts and removed from the final set."

References

1. Pallotti, S. *et al.* Long-read metagenomics reveals stable resistome and microbiome in treated Italian slaughterhouse wastewater: a preliminary study. *Microbiol. Spectr.* (2026) doi:10.1128/spectrum.01562-26.
2. Bellankimath, A. B. *et al.* Culture and amplification-free nanopore sequencing for rapid detection of pathogens and antimicrobial resistance genes from urine. *Eur. J. Clin. Microbiol. Infect. Dis.* **43**, 2177–2190 (2024).
3. Abi Younes, J. N. *et al.* Evaluating the diagnostic performance of long-read metagenomic sequencing compared to culture and antimicrobial susceptibility testing for detection of bovine respiratory bacteria and indicators of antimicrobial resistance. *Antibiotics (Basel)* **14**, 1114 (2025).

Reviewer #1 (Remarks to the Author):

General comment

I would like to thank the authors for addressing all my comments thoroughly. I have only slightly minor comments about Figure 2.

Thank you for reviewing our manuscript. We appreciate your time and valuable feedback on our work.

Minor Comments.

1. Figure 2 labels are barely readable.

We thank the reviewer for pointing it out. Based on the suggestion, we have modified the figure in the updated manuscript.

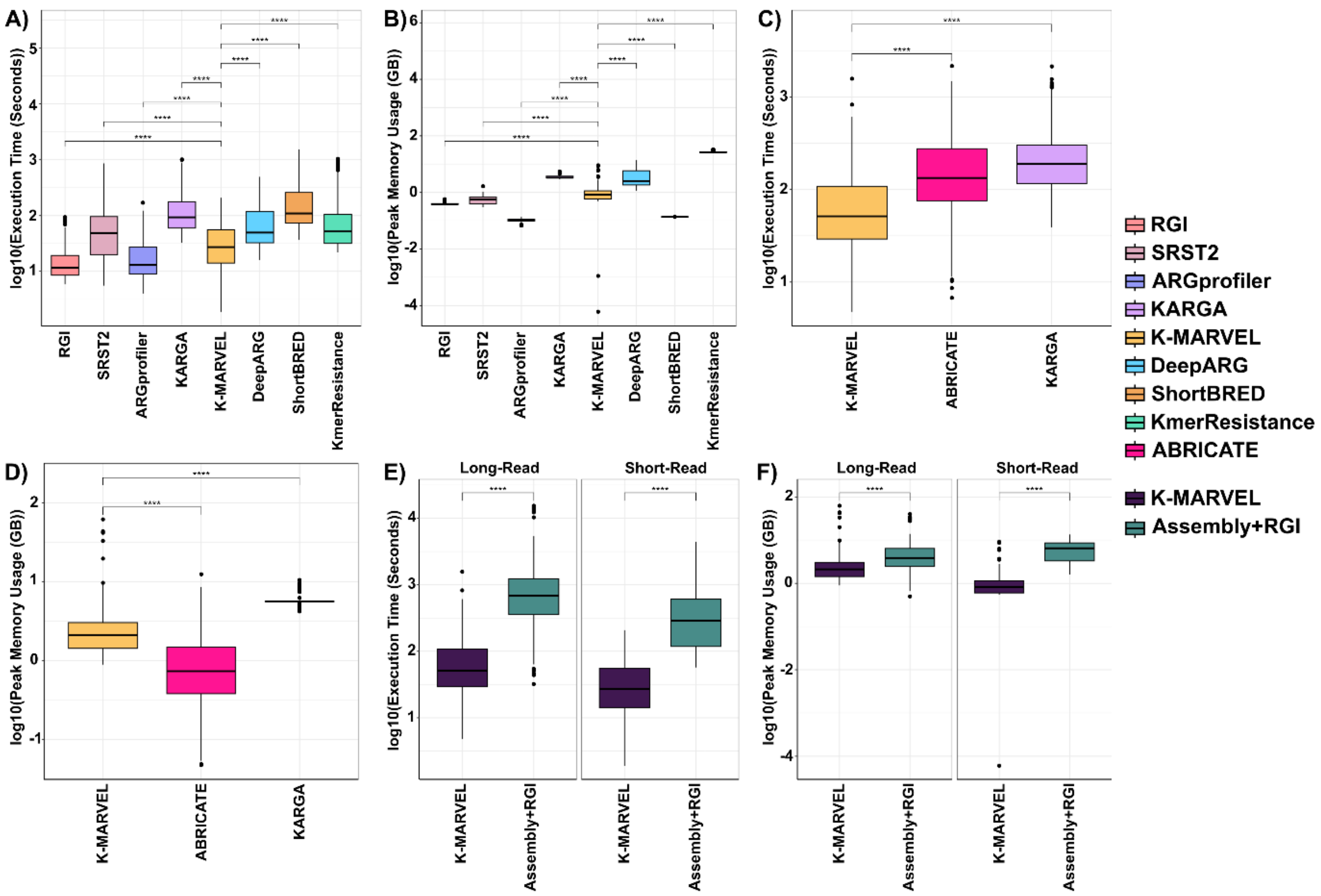

2. Figure 2: Some of the colors don't show in boxes of some subpanels (e.g., subpanel B). I would consider adding a legend in the x-axis labelling the tool corresponding to each box.

Thank you for your suggestion. Based on your suggestion, we have incorporated the required changes in the updated manuscript.

3. Remarks on code availability: The authors have addressed all my comments.

Thank you for testing our tool extensively. We appreciate your time and valuable feedback on our work.
