# Supplementary material for: K-MARVEL: K-Mer-based antimicrobial resistance virtual exploration lab
Source: Nat Commun. 2026 Sep 3;17:10505. doi: 10.1038/s41467-026-77438-8 (PMC13639074; doi:10.1038/s41467-026-77438-8)
Supplement: Supplementary file 3 [file 41467_2026_77438_MOESM1_ESM.pdf]

K-MARVEL: K-Mer-based **A**ntimicrobial **R**esistance **V**irtual **E**xploration **L**ab

Nirmal Singh Mahar[1], Sverre Branders[2], Manfred G. Grabherr[2], Ishaan Gupta[1*], Rafi Ahmad[2*]

[1]Department of Biochemical Engineering and Biotechnology, Indian Institute of Technology Delhi, New Delhi, India-110016

[2]Department of Biotechnology, University of Inland Norway, Holsetgata 22, 2317, Hamar, Norway.

*Corresponding Author

Email: bez207518@iitd.ac.in, sverre.branders@inn.no, manfred.grabherr@inn.no, ishaan@dbeb.iitd.ac.in*, rafi.ahmad@inn.no*

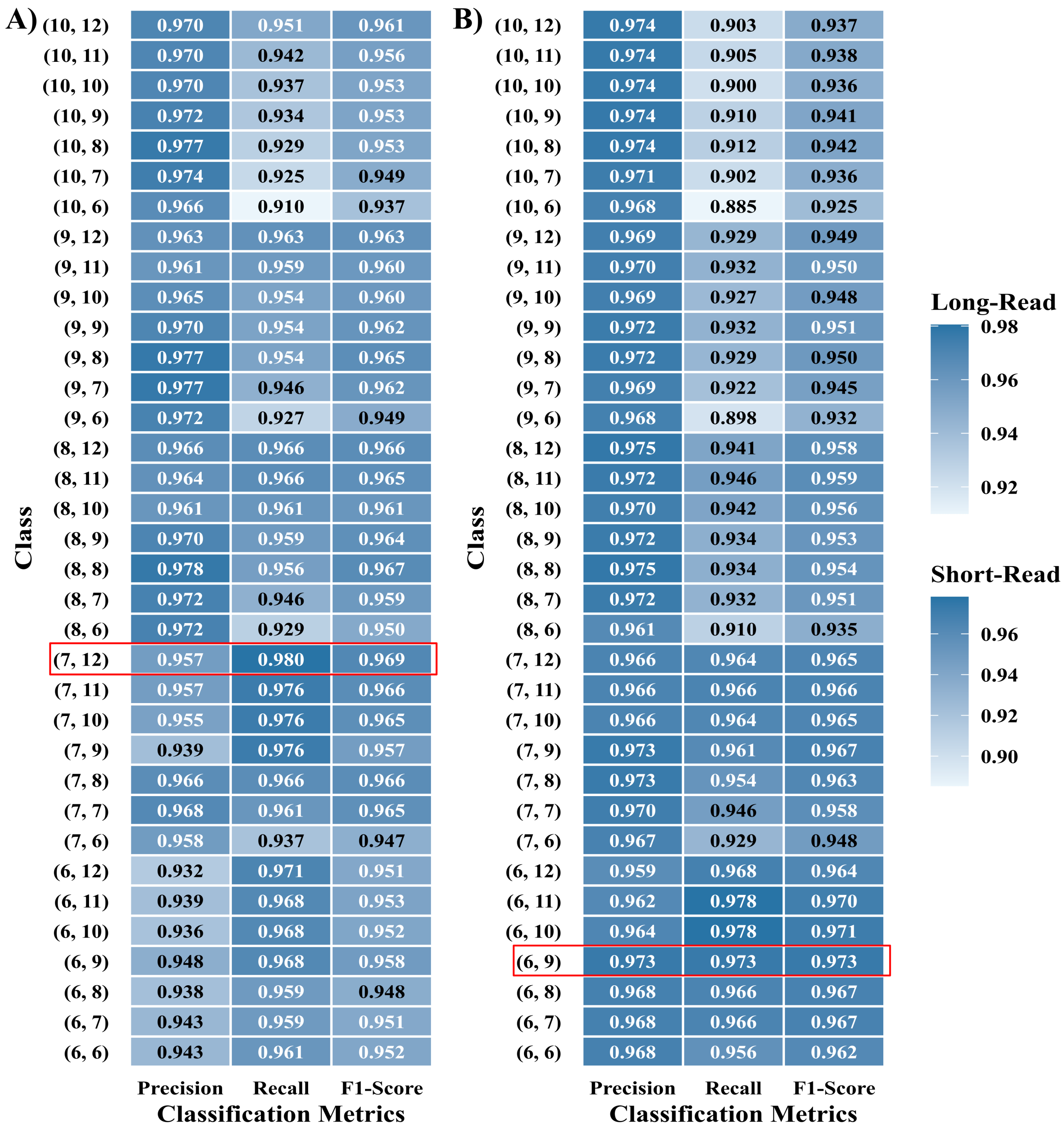

Supplementary Figure 1: Heatmap of classification performance metrics across different combinations of k-mer and extension size used for running K-MARVEL across twelve datasets. **A)** Long read sequencing datasets (n=12). **B)** Short read sequencing datasets (n=12). The highlighted block represents the best combination of k-mer and extension size with the highest F1 score. K-mer size of 7 and extension size of 12 was found to be the best combination for long-read sequencing data, and 6 and 9 for short-read sequencing data.

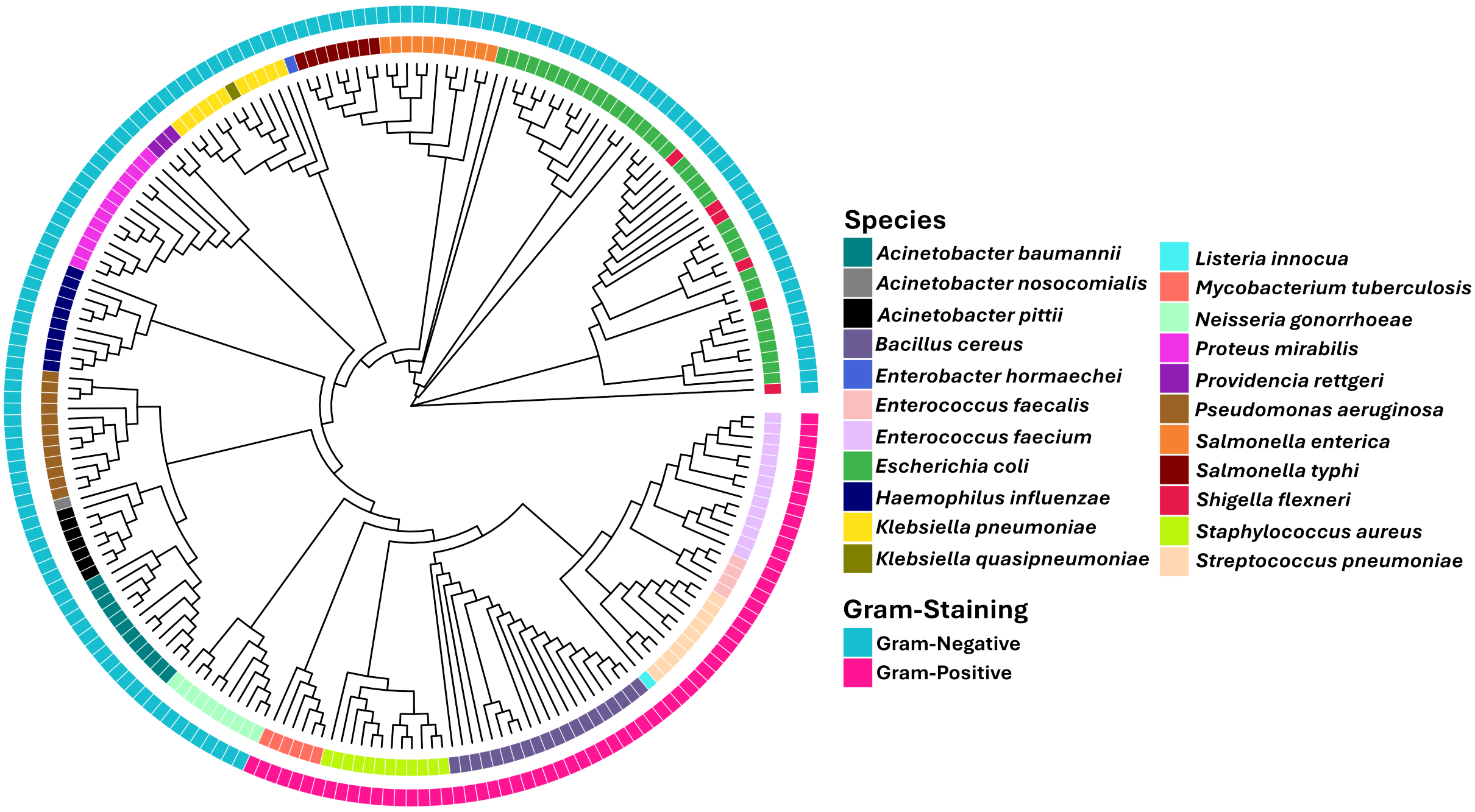

Supplementary Figure 2: Phylogenetic tree illustrating the distribution of bacterial species included in the benchmarking datasets. Most of the species are a part of the pathogen priority list released by the WHO.

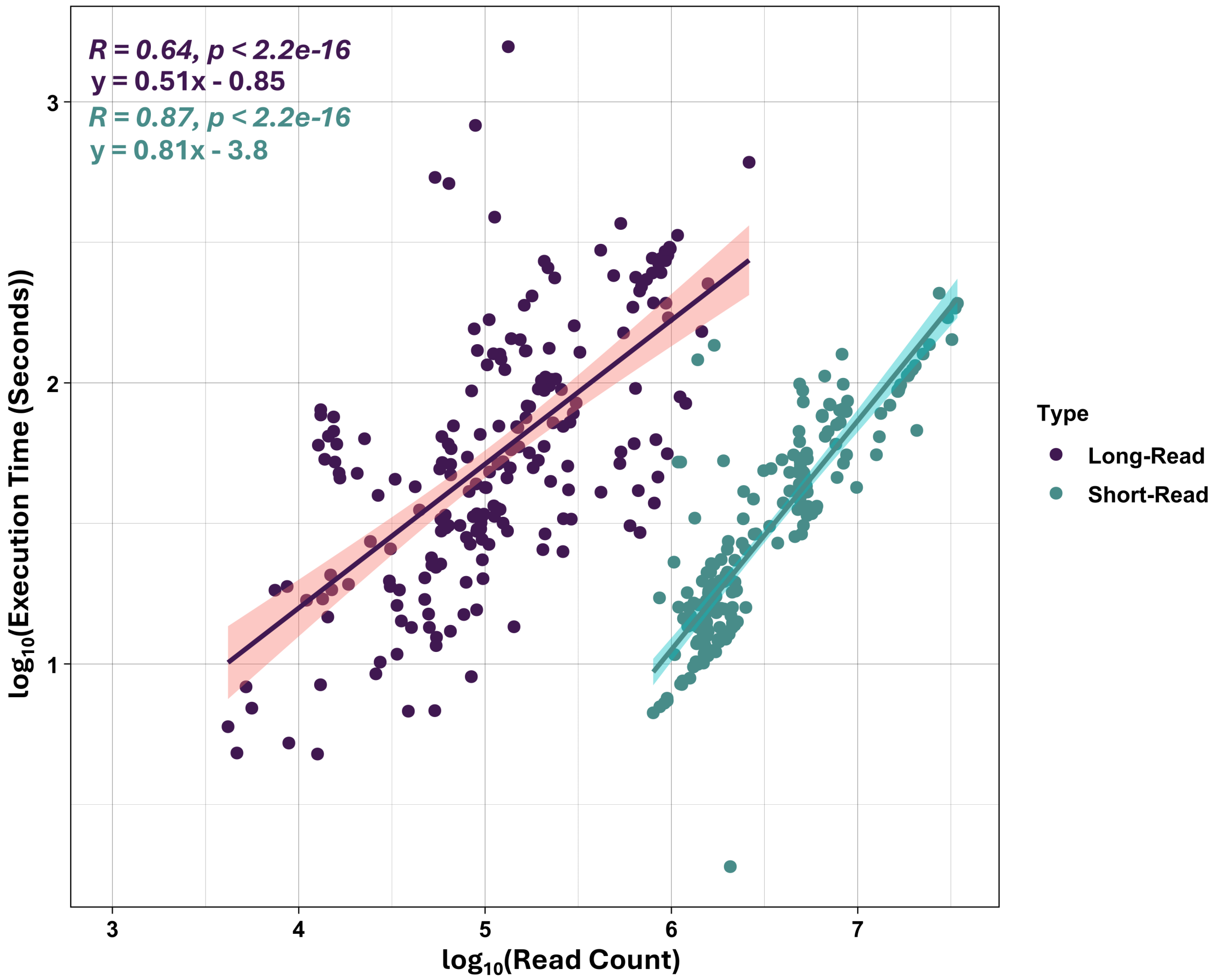

Supplementary Figure 3: Computational performance of K-MARVEL. Run-time was recorded across diverse short-read (teal circles) and long-read (purple triangles) datasets, with both axes $\log_{10}$-transformed to visualize performance across several orders of magnitude of sequence data. Pearson's R (two-sided) was calculated to assess the relationship between the execution time and the number of reads, with multiple testing correction via the Benjamini-Hochberg (BH) procedure. For long-read datasets (n = 209), the adjusted p-value was 9.85×10^{-26} (95% CI: 0.553–0.714), and for short-read datasets (n = 205), the adjusted p-value was 2.88×10^{-64} (95% CI: 0.831–0.899). The execution time scaled near-linearly for both short and long-read datasets, showing the tool's scalability. While the correlation was statistically significant, we observed greater variance in runtime for long-read datasets, which is likely attributed to the read length agnostic technology.

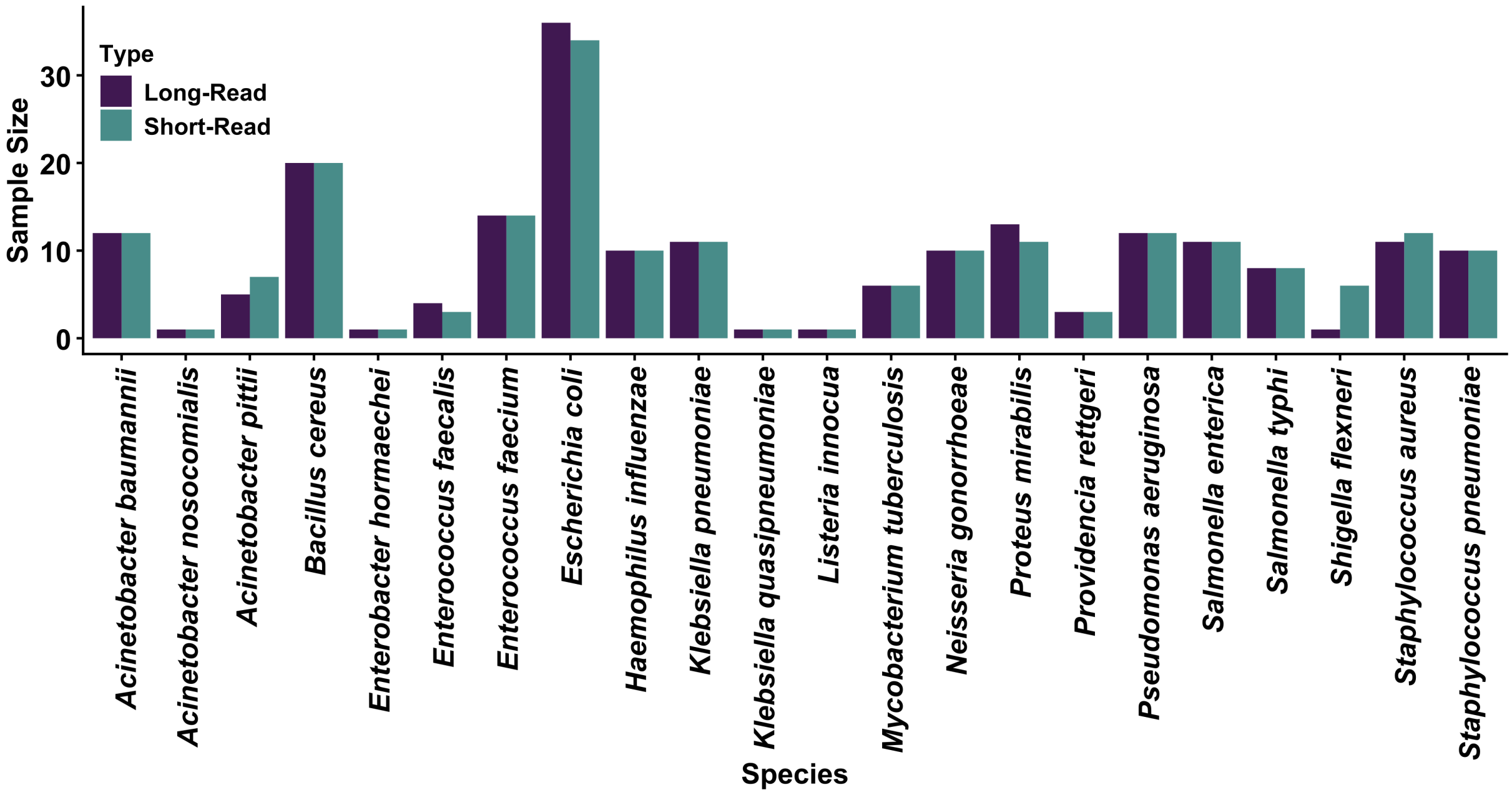

Supplementary Figure 4: Grouped bar plot showing the number of samples per species for each sequencing technology. The plot showcases the breadth of species covered in the testing of K-MARVEL, the majority of which are part of the pathogen priority list released by the W.H.O.
