# Supplementary material for: K-MARVEL: K-Mer-based antimicrobial resistance virtual exploration lab
Source: Nat Commun. 2026 Sep 3;17:10505. doi: 10.1038/s41467-026-77438-8 (PMC13639074; doi:10.1038/s41467-026-77438-8)
Supplement: Supplementary file 4 [file 41467_2026_77438_MOESM2_ESM.pdf]

K-MARVEL: K-Mer-based **A**ntimicrobial **R**esistance **V**irtual **E**xploration **L**ab

Nirmal Singh Mahar[1], Sverre Branders[2], Manfred G. Grabherr[2], Ishaan Gupta[1*], Rafi Ahmad[2*]

[1]Department of Biochemical Engineering and Biotechnology, Indian Institute of Technology Delhi, New Delhi, India-110016

[2]Department of Biotechnology, University of Inland Norway, Holsetgata 22, 2317, Hamar, Norway.

*Corresponding Author

Email: bez207518@iitd.ac.in, sverre.branders@inn.no, manfred.grabherr@inn.no, ishaan@dbeb.iitd.ac.in*, rafi.ahmad@inn.no*

Supplementary Data 1: Detailed information regarding the ground truth, kmer and extension length optimization for both short- and long-read datasets for K-MARVEL.

Supplementary Data 2: Detailed information regarding the datasets used, ground truth, and ARGs capture by K-MARVEL for short-read sequencing datasets.

Supplementary Data 3: Detailed information regarding the datasets used, ground truth, and ARGs capture by K-MARVEL for long-read sequencing datasets.

Supplementary Data 4: Detailed classification metrics for each tested tool, across each bacterial species and sequencing technology.

Supplementary Data 5: Detailed information on the ground truth and ARGs captured by each tested tool for short-read sequencing datasets.

Supplementary Data 6: Detailed information on the ground truth and ARGs captured by each tested tool for long-read sequencing datasets.

Supplementary Data 7: Detailed information about the false positives and negatives categorized based on sequencing technology for ARGs categorized under the protein variant model and protein overexpression model.

Supplementary Data 8: Detailed information about the execution time, peak memory usage, and other computation metrics for K-MARVEL and other tested methods for both sequencing technologies.

Supplementary Data 9: Detailed information regarding the classification performance of K-MARVEL on duplex long-read datasets.

Supplementary Data 10: Detailed information regarding the publicly available datasets utilized for the testing of K-MARVEL.
